# Impaired glutamylation of RPGR^ORF15^ underlies the cone-dominated phenotype associated with truncating distal ORF15 variants

**DOI:** 10.1073/pnas.2208707119

**Published:** 2022-11-29

**Authors:** Jasmina Cehajic-Kapetanovic, Cristina Martinez-Fernandez de la Camara, Johannes Birtel, Salwah Rehman, Michelle E. McClements, Peter Charbel Issa, Andrew J Lotery, Robert E. MacLaren

**Affiliations:** ^a^Nuffield Laboratory of Ophthalmology, Department of Clinical Neurosciences, John Radcliffe Hospital, Level 5 & 6, West Wing OX3 9DU, United Kingdom; ^b^Oxford Eye Hospital, Oxford University Hospitals The National Health Service Trust, John Radcliffe Hospital, West Wing OX3 9DU, United Kingdom; ^c^Department of Ophthalmology, University of Bonn, 53127 Bonn, Germany; ^d^Clinical Neurosciences Research Group, Clinical and Experimental Sciences, Faculty of Medicine, University of Southampton, SO16 6YD Southampton, United Kingdom; ^e^University Hospital Southampton NHS Foundation Trust, SO16 6YD Southampton, United Kingdom

**Keywords:** RPGR, TTLL5, cone dystrophy, gene therapy, glutamylation

## Abstract

Pathogenic variants in the *Retinitis pigmentosa GTPase regulator (RPGR)* gene lead to a clinically severe form of X-linked retinal dystrophy. However, it remains unclear why some variants cause a predominant rod, while others result in a cone-dominated phenotype. Post-translational glutamylation of the photoreceptor-specific RPGR^ORF15^ isoform by the TTLL5 enzyme is essential for its optimal function in photoreceptors, and loss of TTLL5 leads to retinal dystrophy with a cone phenotype. Here we show that RPGR retinal disease, studied in a single cohort of 116 male patients, leads to a clear progressive shift from rod- to cone-dominating phenotype as the RPGR^ORF15^ variant location approaches the distal part of the Open Reading Frame 15 (ORF15) region. The rod photoreceptor involvement on the contrary diminishes along the RGPR sequence, and the variants associated with the cone only phenotype are located predominantly in the very distal part, including the C-terminal basic domain. Moreover, these distal truncating RPGR^ORF15^ variants disrupt the interaction with TTLL5 and lead to a significant impairment of RPGR glutamylation. Thus, consistent with the phenotype of TTLL5 pathogenic variants, our study shows that RPGR^ORF15^ variants, which disrupt its basic domain and the interaction with TTLL5, also impair RPGR glutamylation and lead to the cone phenotype. This has implications for ongoing gene therapy clinical trials where the application of RPGR with impaired glutamylation may be less effective in treating RGPR dystrophies and may even convert a rod–cone dystrophy into a cone dystrophy phenotype.

## Significance

*Retinitis pigmentosa GTPase regulator (RPGR)* disease variants lead to considerable phenotypic heterogeneity in terms of relative involvement of rod and cone photoreceptors. We have identified a clear progressive shift from rod- to cone-dominating phenotypes as the variants move toward the distal end of the gene. Moreover, we find that truncating distal RPGR^ORF15^ variants impair RPGR glutamylation, mediated by TTLL5 enzyme, mirroring the cone-dominated phenotype associated with loss-of-function mutations in TTLL5. The application of RPGR with impaired glutamylation in retinal gene therapy may be less effective in treating RPGR dystrophies.

*Retinitis pigmentosa GTPase regulator* (*RPGR*)-related retinal disease is the commonest form of X-linked and recessive retinitis pigmentosa (RP) accounting for approximately 20% of all RP cases (1, 2). The phenotype is characterized by early onset severe photoreceptor degeneration with rapid progression to blindness, making it a focus of many preclinical and clinical studies aimed at developing genetic therapies (3–5). There is marked phenotypic heterogeneity where mutations in this single gene give rise to phenotypes that predominantly affect the rods (the rod–cone phenotype) and the cones (the cone–rod phenotype) or are associated with foveal or macular atrophy (the cone phenotype). The RPGR genotype is equally heterogeneous and disease-causing variants have been identified in the shared exons (exons 1–14) of the two commonest isoforms*, RPGR^Ex1–19^* and *RPGR^ORF15^*, and in the exon *ORF15* specific to the *RPGR^ORF15^*, while none have been linked to exons 16–19 of *RPGR^Ex1–19^*. Currently, more than 500 pathogenic variants have been identified throughout the length of the photoreceptor specific *RPGR^ORF15^* isoform (*Human Genome Mutation Database, HGMD*) and over 55% of these variants occur within the *ORF15* region. RPGR disease pathogenesis, however, remains poorly understood and to date there is no molecular mechanism to explain why some mutations lead to the predominant rod dystrophy whilst others result in the predominant cone phenotype. The challenge in this is the largely undetermined and complex RPGR structure–function relationship in photoreceptors.

*RPGR*, located on the X chromosome, is alternatively spliced and numerous protein coding transcripts are expressed in many tissues throughout the body. As mentioned above, the two major isoforms are *RPGR^Ex1–19^* (815 amino acids) which is constitutively expressed including the retina, and RPGR^ORF15^ (1,152 amino acids) which is a unique isoform primarily expressed in the connecting cilia of retinal photoreceptors. Its N-terminal domain is the same as in the constitutive isoform, RPGR^Ex1–19^, and it is homologous to the regulator of chromosome condensation 1 (RCC1), known as the RCC1-like domain. This N-terminal domain is followed by the region encoded by the ORF15 consisting of 567 amino acids ordered in highly repetitive tandems of glutamate and glycine residues (Glu-Gly-rich domain) and the nonrepetitive and highly conserved basic domain, rich in basic amino acids at the C terminus (6). While the function of the RCC1-like domain has been proven to be the regulation of small GTPases such as RAB8A (7), the contribution of the C-terminal half of RPGR^ORF15^ to the photoreceptors maintenance and function is yet to be determined. The photoreceptor connecting cilia, analogous to other nonmotile cilia, represents the connection between the inner and the outer segments and acts as a coordination center of multiple developmental and homeostatic signaling pathways (8, 9). Structurally, it consists of the basal body in the inner segment side and the axoneme, which arises from the basal body through the transition zone and extends up to the outer segments. These structures contain microtubules which are associated to a rich proteome with hundreds of different proteins involved in protein trafficking, structure, and signal transduction (10, 11). The diversity in cellular microtubules and the large diversity of functions that their associated proteins develop are mechanistically controlled by a remarkable number of post-translational modifications that affect the dynamics and organization of the microtubules, and their interaction with other cellular components (12, 13). Glutamylation is one of the essential post-translational modifications that consists in the addition of glutamate side chains onto the gamma carboxyl group of glutamate residues, and it is catalyzed by Tubulin Tyrosine Ligase-like (TTLL) enzymes. The addition of these negatively charged groups to the tubulin in the microtubules surface regulates their interaction with other proteins such as microtubule-associated proteins and molecular motors that use microtubules for tracks (14, 15). RPGR^ORF15^ is part of the connecting cilium proteome where it interacts with other proteins involved in cilia regulatory signaling pathways (11, 16). The protein domains encoded by the ORF15 region specifically interact with multiple proteins, enabling efficient trafficking of selected proteins to the photoreceptor outer segments. The tubulin glutamylase TTLL5 is one of the proteins that interacts with the C-terminal domain of RPGR^ORF15^. Upon interaction with the basic domain, the TTLL core domain glutamylates RPGR^ORF15^ protein by adding negatively charged glutamates to the 11 glutamate-rich consensus motifs located in the Glu-Gly-rich domain (17, 18). Somewhat similar to what occurs with microtubules, glutamylation of RPGR^ORF15^ is likely to affect its stabilization and folding, and its interaction with other proteins, acting as a fine regulator of the RPGR^ORF15^ function.

To explore the RPGR genotype–phenotype relationship, we studied 116 patients with RPGR retinal dystrophy—the largest reported RPGR patient cohort to date. Previous genetic studies have looked at differences in phenotypic severity between ORF15 and exon 1–14 variants with conflicting results. Some studies have reported more severe phenotype associated with ORF15 variants (19, 20), some have shown the opposite effect (2, 21), yet others have demonstrated no clear genotype–phenotype relationship (22). Other studies have looked at the nature of RPGR phenotypes noting that variants in exons 1–14 have tendency to result in a rod–cone phenotype, whereas the distal ORF15 variants have more of a cone–rod or cone phenotype (1, 2, 19, 23–28). None, however, has uncovered the underlying mechanism. In this study, we demonstrate a clear shift from rod- to cone-dominated phenotype as the variant location moved along the *RPGR^ORF15^* gene, from N to the C terminus. We show that the distal truncating variants associated with the cone-dominated RPGR phenotype disrupt the basic domain and affect interaction with the TTLL5 enzyme in a PLA. The truncating variants, which express almost full-length *RPGR^ORF15^*protein, also impair *RPGR^ORF15^* glutamylation, mirroring the cone phenotype associated with the loss-of-function TTLL5-related retinal dystrophy (29, 30).

## Results

### RPGR Retinal Disease Displays Marked Genotypic and Phenotypic Heterogeneity.

**Fig. 1.**
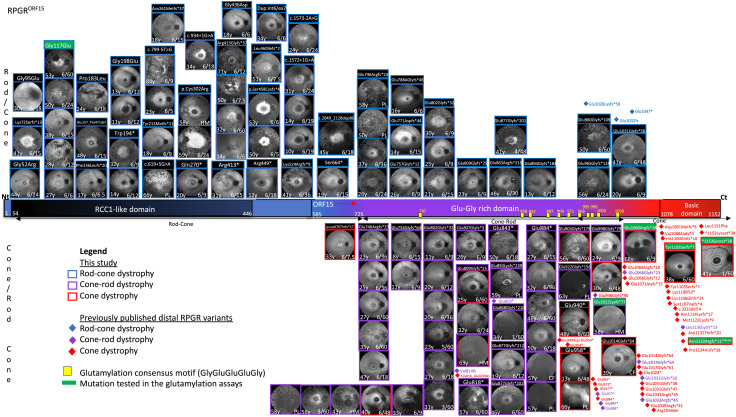
RPGR variants lead to heterogeneous phenotype while the distal ORF15 variants predominantly cause cone dystrophies. Schematic of the structure of RPGR^ORF15^ isoform showing key domains: RCC1-like domain, Glu-Gly-rich domain and basic domain, and glutamylation consensus motifs (yellow rectangles). Fundus autofluorescence images from 108 male patients are shown according to the location of the disease-causing mutation along the RPGR sequence. Patients carried genetically confirmed RPGR variants (denoted on top of each image) which led to four predominant phenotypes: rod–cone (blue frame, top of the RPGR), cone–rod (purple frame, bottom of the RPGR), mixed cone and cone–rod (purple/red frame, bottom of the RPGR), and cone dystrophy (red frame, bottom of the RPGR). Previously published distal RPGR variants leading to disease are also shown including rod–cone (blue diamonds), cone–rod (purple diamonds), and cone dystrophy (red diamonds). Variants tested in the glutamylation assay in this study are highlighted in green.

Previous studies have shown a mutational hot spot within ORF15, the 3’ region which is unique to RPGR^ORF15^ (31, 32). The open-reading frame includes exon 15 and a section of intron 15 made up of highly repetitive purine-rich nucleotides which are inherently unstable and prone to coding errors within the resulting glycine and glutamic acid repetitive domain. Since then, more widespread use of next-generation sequencing has increased the overall pick-up rate of RPGR variants; however, only very few laboratories have optimized the sequencing for ORF15 analysis. In this study, RPGR variants were detected in 116 male patients and recorded on our genetic databases. A total of 60 different pathogenic variants were identified throughout the length of the photoreceptor specific RPGR^ORF15^ (1,152 amino acids). The disease variants leading to a heterogeneous RPGR phenotype are represented in Fig. 1. We found the vast majority of variants to be truncating (45/60 = 75%; 35 frameshifts (31 small deletions, two larger deletions, one small duplication and one small indel) and 10 nonsense), followed by missense changes (7/60 = 12%; one substitution c.3458A>C led to extension p.*1153Serext*38), splice-site variants (5/60 = 8%), two large duplications (2/60 = 3%) and one gross deletion (1/60 = 2%). The most frequent variant was a frameshifted truncation c.2405_2406delAG p.Glu802Glyfs*32 found in 15 patients, followed by c.2236_2237delGA p.Glu746Argfs*23 in nine affected cases. The commonest missense variant was c.350G>A p.Gly117Glu in six individuals. Consistent with previous studies (31, 32), we found that the terminal exon ORF15, which makes up approximately 50% of the total RPGR protein length (encoding 567 amino acids from c.1754 to 3459), harbored close to 60% of pathogenic variants (34/60 = 57%) in 74 patients. Most variants here were truncating (32/34 = 94%) with 26 frameshifts (of which 24 were small deletions, one larger deletion and one indel) and six nonsense variants; one large duplication and one substitution–extension variant. There were no missense variants in ORF15 region. The most difficult to sequence central part (c.2470–3230 encoding for 253 aa) comprised 21 variants (21/60 = 35%) in 30 patients. Two variants, a small truncation and an extension, in two cases were found at the beginning of the basic domain (2/60 = 3%). Exons 1–14 (encoding 584 initial amino acids) harbored 26 pathogenic variants (26/60 = 43%) in 42 patients, 19 of which were found in the RCC1-like domain (c.162-1338, exons 3–10) (19/60 = 32%) in 32 patients. In contrast to ORF15, truncating variants were less frequent and accounted for 46% (12/26 variants) of the total exon 1–14 variants. 8 truncating variants were frameshifts—all small deletions, and four were nonsense variants. All missense variants were located here, outside the ORF15 region (7/26 = 27%). In addition, there were 5/26 = 19% splice-site variants, one gross deletion and one gross duplication in exons 1–14.

**Fig. 2.**
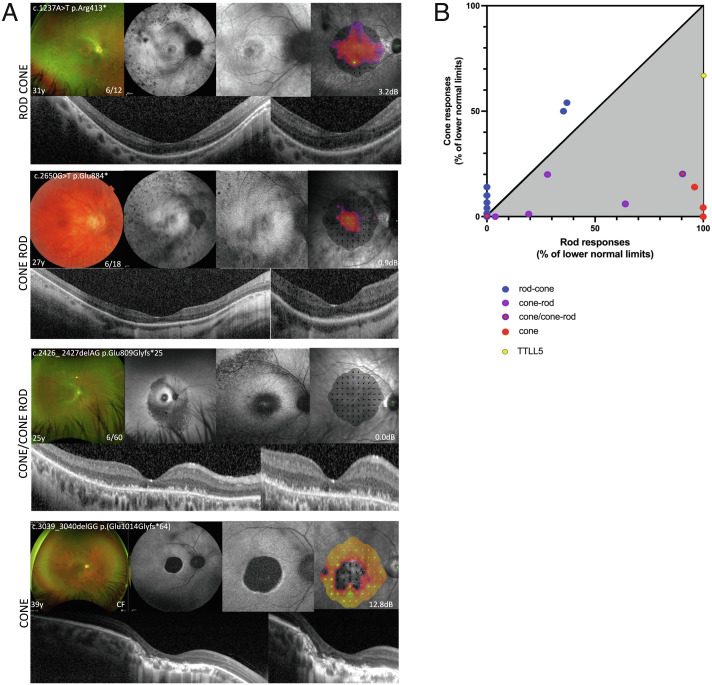
RPGR disease leads to four distinct phenotype groups: rod–cone, cone–rod, cone–cone–rod, and cone dystrophy. Retinal imaging and microperimetry studies (*A*) and electrodiagnostic studies (*B*). (*A*) RPGR disease causes degeneration of both rods and cones, but within the disease spectrum, we identified four distinct phenotypic groups, albeit with some overlap. The rod–cone dystrophy predominantly affects rods first, with progressive involvement of cones as the disease progresses. On the reverse, cone–rod phenotype predominantly affects cones first with variable involvement of rods with disease advancement. The mixed cone–cone–rod phenotype predominantly affects central cones, but mid-peripheral cones and rods are also affected from early disease. The cone dystrophy phenotype predominantly affects central cones leading to marked macular atrophy as the disease progresses. (*B*) The relationship of rod and cone responses to the lower normal limits on electrophysiology testing. Rod–cone phenotype (blue dots) demonstrates predominant rod system involvement, whereas cone-dominated phenotypes (cone–rod (purple dots), cone–cone–rod (purple/red dots) and cone (red dots)) confirm predominant cone involvement. A patient with a *TTLL5* associated cone dystrophy (yellow dot) is also shown confirming predominant cone involvement on electrophysiology testing, suggesting that the two genes share a similar cone-dominated phenotype. Oblique line, equal reduction of rod and cone amplitudes. Gray area, greater cone than rod involvement.

Previous studies have reported several RPGR-related disease patterns including rod–cone, cone–rod, cone, or macular atrophy based on electrodiagnostic findings in early studies (2, 33) and on clinical phenotyping and imaging in more recent studies (19, 34–36). Herein a total of 108 male patients (age 8–84 y) from the genetic database of RPGR variants underwent deep clinical phenotyping and diagnostic imaging (Fig. 1). We found the commonest form of the RPGR-related disease to be the rod–cone phenotype, also known as the “classic” X-linked RP, present in 56% of 60 patients (60/108) (Fig. 1, blue frames) compared to 94% (22), 85% (37), or 70% (19) of the total number of patients observed in these studies. These patients presented in early childhood with night vision problems and difficulties with dark adaptation due to early loss of function and/or degeneration of peripheral rods. Clinical phenotyping and multimodal retinal imaging (Fig. 2*A*) showed extensive pigmentary retinopathy in the periphery of the retina (wide-field optos images), variable degree of hypo-autofluorescent atrophic patches in mid-periphery which increased with disease progression (*SI Appendix*, Fig. S1*A*). At early stages, cone degeneration was mild, as delineated by hyper-autofluorescent rings encompassing most of the macular area and which constricted with more advanced disease (*SI Appendix*, Fig. S1*A*). This was also evident on grossly preserved central outer retinal structures including the ellipsoid zone (OCT images). Central retinal sensitivity was reduced even at early stages of the disease (microperimetry plots), whereas visual acuity remained preserved. Later, progressive cone degeneration and dysfunction occurred with more severe loss of central retinal sensitivity and reduction in visual acuity in most cases. Thirty-five patients (35/108 = 32%) in our cohort were diagnosed with the cone–rod dystrophy phenotype (Fig. 1, purple frames), compared with 23% (19), 15% (37) or 4% (22) in previous studies. Patients presented with day-time vision problems, photophobia, dyschromatopsia and blurred vision related to cone dysfunction. Phenotype analysis (Fig. 2*A*) showed minimal peripheral pigmentary retinopathy compared to the age-matched rod–cone phenotype. Cone-related degeneration was however more pronounced as evident by central macular hypo-autofluorescence, disrupted ellipsoid zone and markedly reduced visual acuity and mean central retinal sensitivity. These elements were present from very early stages of the disease, and were more pronounced in patients with advanced disease (*SI Appendix*, Fig. S1*B*). Large-area hyper-autofluorescent rings, delineating a transition zone of diseased retina centrally and unaffected retina outside the rings, were evident in childhood (*SI Appendix*, Fig. S1*B*) and initially increased in size with disease progression. Peripheral rod degeneration remained generally mild to moderate, with minimal peripheral pigmentary retinopathy even at very advanced disease stages. Depending on topography of degeneration, we also found that five out of 108 patients developed a mixed cone–cone–rod phenotype predominantly affecting central cones but mid-peripheral cones and rods in a sectoral pattern were also affected from early disease (Fig. 1, purple/red frames). The phenotype was also reported in another study (19), although not categorized as such. Patients with this mixed phenotype presented with central visual problems and mid-peripheral visual field defects in early adulthood, at a slightly later age compared to initial symptoms in rod–cone or cone–rod phenotypes (*SI Appendix*, Fig. S1*C*). Detailed clinical phenotyping (Fig. 2*A*) showed cone-related central degeneration as for the cone–rod phenotype, but central foveal cones tended to be more severely affected. In addition, there was mid-peripheral or sectoral photoreceptor degeneration as shown by marked hypo-autofluorescence. Later, there was progressive macular atrophy (foveal sparing in some cases), central reduction in retinal sensitivity and in some late-stage cases pigmentary retinopathy. Eight out of 108 (7%) patients had a cone dystrophy phenotype (Fig. 1, red frames). The proportion of patients with a cone dystrophy phenotype was similar to one reported study (7%) with the majority of RPGR cone dystrophy cases described via isolated case reports in association with a few rare variants (19). Patients generally had a more delayed onset of presenting symptoms which consisted of difficulties with near and reading vision, but no peripheral visual impairment, nyctalopia, or visual field loss. Phenotype analysis (Fig. 2*A*) demonstrated no peripheral pigmentation, hyper-autofluorescent rings centered at the fovea and encircling an area of profound foveal hypo-autofluorescence, a central scotoma on microperimetry, reduced vision and grossly disrupted central outer nuclear layer on OCT scanning. There was no obvious evidence of rod-related degeneration, or peripheral pigmentary retinopathy even at late-stage macular degeneration (*SI Appendix*, Fig. S1*D*), confirming previous findings (38). However, retinal sensitivity was reduced in the area surrounding the macular atrophy, suggesting a more generalized cone dysfunction.

The use of electrophysiology in characterization of retinal dystrophy phenotypes has diminished substantially over the last few decades and most retinal specialist centers do not perform the studies on routine basis, but instead focus on genetic testing and clinical phenotyping as described above. Except for a few particular genes (e.g., *CACNA1F* (1) and *NR2E3* (39) where the electrophysiology can be almost diagnostic, the value of Electroretinography (ERG) testing to verify the diagnosis remains low. In addition, for many patients the photoreceptor degeneration is already advanced enough at the time of presentation to result in flat or undetectable ERG responses. However, since neither microperimetry nor autofluorescence studies are cone-specific, the full-field ERG is valuable in defining the spectrum of cone and rod involvement in retinal dystrophies, especially in research settings or in cases of unknown genotype and with variants of uncertain pathogenicity. In RPGR, in young patients and in those with mild phenotypes, the relative contribution of rod- and cone-driven ERG responses can be used to distinguish between rod-dominated and cone-dominated phenotypes. We collected ERGs from 25 patients from representative RPGR phenotype groups: rod–cone (n = 12), cone–rod (n = 8), cone–cone–rod (n = 2), and cone (n = 3) (*SI Appendix*, Table S1 and Fig. 2*B*). In rod–cone group (Fig. 2*B*, blue dots, n = 12), scotopic and photopic responses were extinguished in five patients indicating severe photoreceptor degeneration. The remaining seven patients demonstrated substantial (35–37% of the lower normal limit, n = 2) or complete (n = 5) reduction of scotopic amplitudes. Photopic amplitudes also showed marked (50 and 54% of the lower normal limit, n = 2) or near complete (2–14% of the lower normal limit, n = 5) reduction. In all cases with measurable responses, scotopic amplitudes were more reduced compared to photopic, confirming the rod-dominated phenotype in this group (Fig. 2*B*, non-shaded area). In addition, photopic 30 Hz flicker responses were detected (albeit at low amplitudes and increased latencies) in 10 out of 12 patients further confirming the presence of cone responses. Nonetheless, ERG testing in this group confirmed early cone involvement as seen on microperimetry testing. We also recorded ERGs from 13 patients from cone-dominated phenotype groups (Fig. 2*B*, shaded area). In cone–rod group (Fig. 2*B*, purple dots, n = 8), ERG responses were absent in three patients; four patients had substantial/nearly complete reduction in both scotopic (4–28% of lower normal limit) and photopic (1.3–20% of lower normal limit) responses (photopic > scotopic) and one patient had near complete loss of photopic responses (6% of lower normal limit) and moderate reduction in scotopic responses (64% of lower normal limit). In addition, six out of eight patients had no detectable responses at 30 Hz flicker (in rod–cone group only two out of 14 were not detectable), and the two who had measurable responses showed markedly abnormal amplitudes and latencies. Pattern ERGs corresponding to the photoreceptor dysfunction/loss in the macula were recorded in five cases and were undetectable in three and showed marked abnormality in two cases, indicating substantial macular involvement in all five cases. Overall, in the cone–rod group, the photopic responses were more reduced than scotopic, confirming the cone-dominated phenotype. We also recorded ERGs in two out of five patients we characterized with mixed cone–cone–rod phenotype (Fig. 2*B*, red/purple dots). Both patients were in advanced stages of the disease, and in one patient we found no measurable ERG responses. The other patient had a milder phenotype with cone predominant involvement (photopic responses 89% of lower normal limit, scotopic 20% of lower normal limit, undetectable 30 Hz flicker). Pattern ERGs were undetectable in both cases demonstrating macular involvement. Lastly, we obtained ERGs from three patients with RPRG cone dystrophy (Fig. 2*B*, red dots). All three patients had nearly complete loss of photopic responses (4–14% of lower normal limit) and normal (100% of lower normal limit, n = 2) and near normal (96% of lower normal limit, n = 1) scotopic responses. In addition, 30 Hz flicker response was not detected in two patients, and it was hardy detectable in one patient. Pattern ERGs were undetectable in all three cases indicating severe macular involvement. These findings confirm predominant cone involvement in the cone dystrophy phenotype group, supporting previous ERG studies (38). Overall, ERG testing of patients from different RPGR phenotypic groups (Fig. 2*B*) confirms our findings from clinical phenotyping and functional microperimetry data.

### Distal RPGR^ORF15^ Variants Predominantly Lead to Cone Dystrophy.

**Fig. 3.**
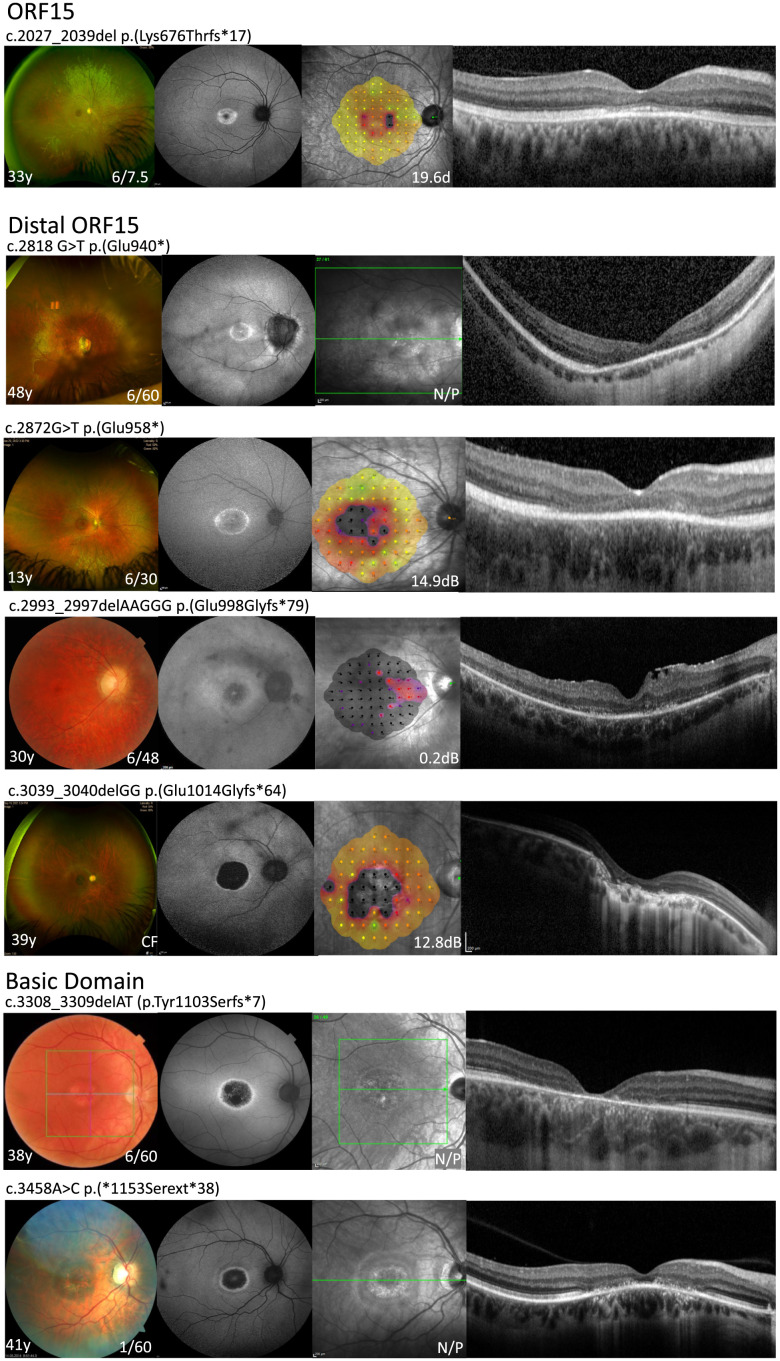
Most cone dystrophies are associated with RPGR^ORF15^ variants at the distal end of the ORF15. The distinct cone dystrophy with central macular atrophy was found in association with seven different variants, mostly located in the very distal part of the ORF15 including both variants within the basic domain. Optos wide-field or fundus color image (first column), fundus autofluorescence (second column), microperimetry (third column, where possible), and horizontal spectral domain OCT (fourth column) are shown for different variants.

Despite the ongoing research on RPGR disease, the genotype–phenotype correlations and the underlying mechanisms remain mostly unresolved (1). In this study, we phenotyped 108 RPGR patients and found a clear shift toward the cone-dominated phenotype as the variants approached the distal end of the RPGR gene (Fig. 1). Exon 1–14 variants, irrespective of the variant type, were exclusively associated with the rod–cone dystrophy (*Top* of Fig. 1), and we found no cases of the other three cone-associated phenotypes. ORF15 variants resulted in a more varied phenotype: rod–cone (42%), cone–rod (48%), mixed cone–cone–rod (15%), and cone dystrophy (21%) with some variants leading to more than one phenotype. For example, four ORF15 variants (12%) namely (c.2236_2237delGA p.Glu746Argfs*23, c.2405_2406delAG p.Glu802Glyfs*32, c.2426_2427delAG p.Glu809Glyfs*25, c.2426_2427delAG p.Glu809Glyfs*25) led to both rod–cone and cone–rod phenotypes and two of these variants (c.2405_2406delAG p.Glu802Glyfs*32, c.2426_2427delAG p.Glu809Glyfs*25) in addition resulted in the mixed cone–cone–rod phenotype. One variant (c.2993_2997delAAGGG p.Glu998Glyfs*79) led to both cone–rod and the cone phenotype. A notable finding is that all cases of predominant cone dystrophies (cone–rod, cone–cone–rod and cone phenotypes) were in the ORF15 region with a clear shift toward the cone-dominated dystrophy phenotype caused by the variants at the C terminus of the gene (Figs. 1 and 3). In fact, the two basic domain variants in our cohort (c.3308_3309delAT p.Tyr1103Serfs*7, c.3458A>C p.*1153Serext*38) and the vast majority of the previously published basic domain variants (Figs. 1 and 3) were cone-dominated dystrophies with central macular atrophy. On the contrary, we found that the effect of disease variants diminished in rod photoreceptors downstream of the distal ORF15, and we found no rod-involving phenotype associated with basic domain variants in our cohort. In addition, most cases recorded in HGMD database to date (with one exception) are cone dystrophies and there are no recorded cases of rod–cone dystrophy associated with basic domain variants.

Overall, our patient cohort unequivocally illustrates a shift in the predominant phenotype, from rod–cone to cone–rod to cone-dominating phenotype, as the variant location moves downstream of *RPGR^ORF15^.*

### RPGR Disease Variants Lead to Early Loss of Cone Function.

**Fig. 4.**
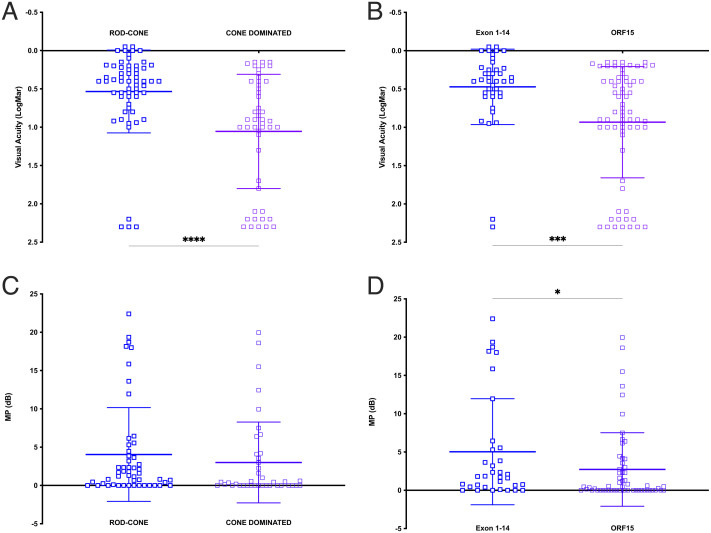
RPGR disease leads to loss of visual function depending on disease phenotype and variant location along the RPGR genome. Mean visual acuity for rod–cone versus cone-dominated phenotype (*A*) or variant location in exons 1–14 versus ORF15 (*B*). Mean microperimetry for rod–cone versus cone-dominated phenotype (*C*) or variant location in exons 1–14 versus ORF15 (*D*). Following tests for normal distribution, significance was tested by Mann–Whitney test (*P < 0.05).

In order to assess the effect of RPGR disease on retinal function, we studied two biomarkers, the best corrected visual acuity (BCVA, logMAR) and the mean mesopic microperimetry (MP, decibels, dB), both of which predominantly assess cone-mediated vision. *SI Appendix*, Fig. S2 shows BCVA and the microperimetry plots (available in 91 cases) for our RPGR patient cohort related to the variant location along the RPGR. There was considerable variation in BCVA among the RPGR subjects related to the variant location along the RPGR genome (*SI Appendix*, Fig. S3*A*) and patients’ age (*SI Appendix*, Fig. S3*B*). The overall cohort trend for the BCVA was to decline as the variants approached the distal end of *RPGR^ORF15^* toward the cone predominant phenotypes (*SI Appendix*, Fig. S3*A*) with significantly higher BCVA associated with rod–cone compared to cone-dominated variants (Fig. 4*A*, *P* < 0.00005) or with variants in exons 1–14 compared to ORF15 variants (Fig. 4*B*, *P* < 0.0005) although the difference between the variants in exons 1–14 and ORF15 was not significant when we looked only at the variants associated with the rod–cone phenotype (*SI Appendix*, Fig. S3*C*). We also found significant differences in BCVA amongst the phenotypic groups with the rod–cone phenotype resulting in best visual acuity followed by the cone phenotypes (*SI Appendix*, Fig. S3*D* rod–cone v cone rod *P* < 0.005 and rod–cone v cone *P* < 0.05). To control for the bias caused by advancing age on disease progression and decline in BCVA (*SI Appendix*, Fig. S3*B*), we also looked at the phenotypic differences in age-matched groups (*SI Appendix*, Fig. S4). We found visual acuity to be mildly affected in rod–cone and cone-dominated dystrophy in age group 0–20 y (except for one case of early cone dystrophy with more severely affected acuity), with no statistical differences (*SI Appendix*, Fig. S4*A*). From then on, visual acuity was more severely reduced in cone-dominated phenotype group with statistical differences at age groups 30–40 y (*P* < 0.05), 40–50 (*P* < 0.0005) and 50 y plus (*P* < 0.05) (*SI Appendix*, Fig. S4*A*). The BCVA was also significantly reduced when associated with ORF15 compared to exon 1–14 variants for age-matched subjects (*SI Appendix*, Fig. S4*B*) at 30–40-y groups (*P* < 0.05) and at 50 plus-year groups (*P* < 0.005).

We found mesopic microperimetry to vary considerably between the subjects depending on variant location along the RPGR genome (*SI Appendix*, Fig. S3*E*) and patients’ age (*SI Appendix*, Fig. S3*F*) There was an overall mild tendency for mean microperimetry to decline as the variants approached the distal end of *RPGR^ORF15^* toward the cone-dominating phenotypes, except in some cases of cone only phenotype where the mean retinal sensitivity remained higher (*SI Appendix*, Fig. S3*E*). Overall, the microperimetry was moderately higher when associated with rod–cone versus cone-dominated phenotypes (Fig. 4*C*) reaching statistical difference when comparing all variants in exons 1–14 and ORF15 (Fig. 4*D*, *P* < 0.05). There was no statistical difference when quantifying only the variants associated with the rod–cone phenotype in exons 1–14 versus ORF15 (*SI Appendix*, Fig. S3*G*). In addition, when mean central retinal sensitivity was analyzed in phenotypic groups (*SI Appendix*, Fig. S3*H*), we found that in five cases of cone only phenotype, the mean microperimetry was higher than the average for other cohorts, but the overall cohort differences were not significant. Moreover, we analyzed microperimetry across the phenotypic groups in age-matched subjects (*SI Appendix*, Fig. S5*A*) and found no overall phenotypic differences in age group 0–20 y. Microperimetry worsened in cone-dominating phenotypes in the age group 20–30 y. However, the mean microperimetry for the two cases of the cone only phenotype at the age group 30–40 y was much higher compared to the other phenotypes, but the overall difference between the cone–dominated and rod–cone phenotype groups remained statistically not significant. When the microperimetry pattern was analyzed in detail, we found sharply demarcated scotomas centered on the fovea corresponding to the central atrophy patch (Fig. 3), with only moderate reduction in sensitivity in the surrounding area. That said, the microperimetry surrounding the atrophic patch was not normal, suggesting a more generalized cone dysfunction. The microperimetry reduced to almost zero in most cases after the age of 40, with a few exceptions in the rod–cone phenotype group with relatively preserved central sensitivity (four out of 14 cases were above the 2.0 dB average in 50 plus year group, *SI Appendix*, Fig. S5*A*) and the one outlier in cone-dominated group with a cone–cone–rod dystrophy. In addition, we found no statistical differences in microperimetry between exons 1–14 and ORF15 variants in age-matched subjects (*SI Appendix*, Fig. S5*B*).

Taken together, our functional studies imply that the cone-mediated vision, as assessed by BCVA and microperimetry become affected early in the RPGR disease process across all phenotype groups. In the age group 0–20, most subjects had only mildly reduced vision (mean BCVA (logMAR) = 0.33 (±0.05) for rod–cone, 0.24 (±0.04) for cone–rod and 0.9 for one cone case). However, despite the near normal visual acuity, even the youngest patients (age 0–20) showed reduced microperimetry (mean microperimetry (dB) was 13.0 (±2.61) for rod–cone, 12.6 (±5.98) for cone–rod and 15.5 for one case of cone only dystrophy (normally expected for this age group is ∼30 dB). Progressive cone involvement in cone–rod or cone dystrophy reduced mean visual acuity and central retinal sensitivity. This effect became more notable after the age of 30 compared to the rod–cone phenotype. The exception to this was the microperimetry in the cone-only phenotype where the average microperimetry, despite the dense central scotoma, was more preserved. Overall, cone dystrophies which were mostly associated with the distal *RPGR^ORF15^*variants led to delayed onset, profound central visual loss, but the microperimetry data also showed that the cone dysfunction was more generalized, reducing retinal sensitivity in the area outside the central scotoma in addition to a total loss of retinal sensitivity corresponding to the area of foveal cone atrophy.

### Truncating Distal RPGR^ORF15^ Variants Associated with Cone Dystrophies Impair RPGR^ORF15^ Interaction with TTLL5.

**Fig. 5.**
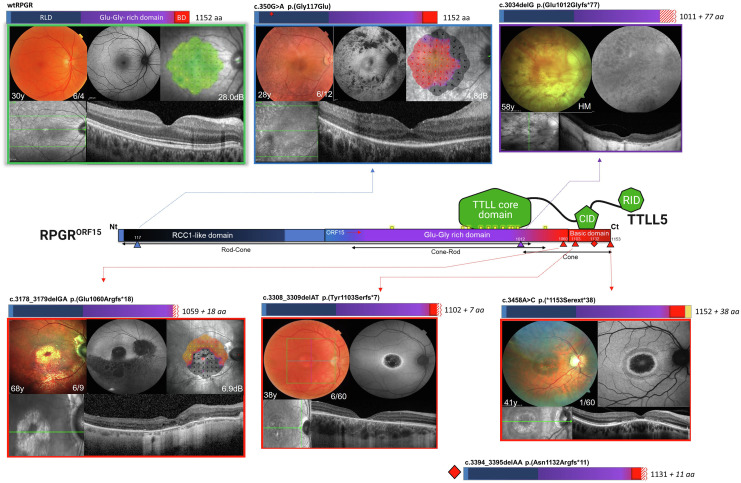
RPGR^ORF15^ is glutamylated in the retina by the enzyme TTLL5, for which the basic domain and the glutamic acid-glycine rich region are required. Schematic diagram of the interaction between RPGR^ORF15^ and TTLL5 is shown in the center of the figure. The cofactor interaction domain (CID) of TTLL5 binds the basic domain of RPGR^ORF15^ and the TTLL core domain initiates the side chain of glutamates on multiple Glu-Gly repeats, which are glutamylation consensus motifs (yellow boxes). The receptor interaction domain (RID) has an unknown function. Representation of the human RPGR^ORF15^ wild type protein (full length, 1,152 amino acids) leading to normal retinal phenotype and the abnormal RPGR proteins analyzed in this study with corresponding disease phenotypes. Predicted RPGR alteration p.Gly117Glu in the N-terminal RCC1-like domain is the result of a single base pair substitution c.350G>A and does not affect the length of the protein or the structure of the C-terminal domain. This missense change results in rod–cone dystrophy. Predicted alterations p.Glu1012Glyfs*77, p.Glu1060Argfs*18, p.Tyr1103Serfs*7, and p.Asn1132Argfs*11 are the result of one or two nucleotide deletions, c.3034delG, c.3178_3179, c.3308_3309delAT, and c.3394_3395delAA respectively which disrupt the natural triplet reading frame of the DNA sequence and results in truncated forms of the protein, which affects the basic domain. The four alterations are pathogenic and result in a cone-related phenotype. Predicted RPGR alteration p.*1153Serext*38 is caused by an *A*–*C* substitution in the last stop codon of the protein c.3458A>C, generating an extension of the wild-type sequence. This change leads to cone dystrophy. Fundus color image (first top column), fundus autofluorescence (second top column), microperimetry (third top column, where possible), and horizontal spectral domain OCT (bottom image) are shown for the wild-type RPGR and for the variants analyzed in interaction and glutamylation assays. Phenotypes: normal (green frame), rod–cone (blue frame), cone–rod (purple frame), and cone–cone–rod (purple/red frame), and cone (red frame).

Considering that the majority of cone dystrophies are associated with pathogenic RPGR variants at the distal end of the ORF15, we hypothesized that the distal part of the protein plays an important role in normal functioning of cone photoreceptors. Post-translational glutamylation of the photoreceptor-specific RPGR^ORF15^ by the TTLL5 enzyme is necessary for its function (17). In the first instance, the TTLL5 needs to interact with ORF15 via the C terminus, in order to glutamylate RPGR (Fig. 5). Following that the loss of the TTLL5 enzyme leads to a cone-dominated phenotype (29, 30), where full-length RPGR would not be glutamylated, we hypothesize that the phenotype of the distal pathogenic RPGR variants would merge with the TTLL5 retinal dystrophy.

Variants of the *TTLL5* gene are a rare cause of retinal dystrophy and to date only a handful of cases have been described in the literature, with two studies in particular (29, 30), detailing clinical and genetic findings. Specifically, both studies report biallelic variants in *TTLL5* causing cone (n = 5) or cone–rod (n = 4) phenotypes and one case of early onset severe retinal dystrophy. In all cases of cone dystrophy, irrespective of age, there was central and peripheral cone dysfunction with preservation of rod photoreceptor function on ERG, much like what we observed with *RPGR* related cone dystrophy. Four subjects with cone–rod phenotype had early severe generalized cone system dysfunction and additional involvement of rods. In addition to these reports, we also identified a patient on our database with biallelic variants (c.1060G>A p.(Val354Met) and c.1441G>A p.(Gly481Arg)) in this rare gene. The patient was a 68-y-old female who presented with photophobia, color vision abnormalities, and vision loss in early childhood. Her visual acuity at age of 68 was severely reduced at 0.9 logMar, yet her anterior segment examination and fundoscopy were unremarkable. Retinal imaging by fundus autofluorescence showed increased hypo-autofluorescence at the fovea and OCT imaging confirmed marked loss of the central ellipsoid zone indicating loss of cone photoreceptors (*SI Appendix*, Fig S6). Electrophysiology recordings demonstrated moderate reduction in amplitude in photopic responses (67% of lower normal limit) and intact scotopic rod responses (100% of lower normal limit) (*SI Appendix*, Table S1 and Fig. 2*B* (yellow dot)). In addition, pattern ERG was not detectable, indicating severe macular pathology. Overall, ERG testing confirmed cone dystrophy phenotype with normal rod-driven responses, similar to responses found in *RPGR* cone dystrophy phenotype, suggesting that the two genes share similar cone-dominated phenotype. Nonetheless, it is feasible that in very advanced cases of cone dystrophy, some rod dysfunction might also occur.

Since the CID of TTLL5 binds the C-terminal basic domain of RPGR^ORF15^ to glutamylate the protein, we analyzed how variants associated with a predominant cone phenotype (cone–rod, mixed cone–cone–rod, or cone dystrophy) located in the distal end of RPGR^ORF15^ affect this interaction (Fig. 5). To assess the physical interaction between the different RPGR variants and TTLL5, we carried out a proximity ligation assay (PLA) followed by the immunodetection of RPGR in transiently transfected HEK293 cells, using an antibody raised against the N-terminal region of RPGR (see *SI Appendix*, *SI Materials and Methods* for more details). The variants analyzed in this study were selected from the three cone-dominated phenotype groups which resulted in progressively shorter sequence truncations or an RPGR protein extension: c.3034delG p.Glu1012Glyfs*77 (87.8% non-altered sequence identical to wild type, 6.7% altered amino acid sequence and 5.5% truncated) (cone–rod phenotype); c.3178_3179delGA p.Glu1060Argfs*18 (92% non-altered sequence identical to wild-type, 1.5% of the mutant is frameshifted and 6.5% truncated) (mixed cone–cone–rod phenotype); c.3308_3309delAT p.Tyr1103Serfs*7 (95.7% non-altered sequence identical to wild type, 0.6% altered amino acid sequence, and 3.7% truncated) (cone phenotype); c.3394_3395delAA p.Asn1132Argfs*11 (98.2% non-altered sequence identical to wild type, 0.9% altered amino acid sequence, and 0.9%. truncated) (cone phenotype) and c.3458A>C p.*1153Serext*38 (100% non-altered sequence identical to wild type plus 3.2% extension) (cone phenotype). First, we performed an in silico prediction using the NMD Esc Predictor (40) to understand whether the truncated variants analyzed in this assay are likely to escape non-sense-mediated decay (NMD) and generate a stable protein. The RPGR transcripts harboring the frameshift mutations specified above are predicted to escape NMD (*SI Appendix*, Fig. S7). Thus, we assume that these mutations result in a stable transcript which is translated into a stable truncated protein product.

**Fig. 6.**
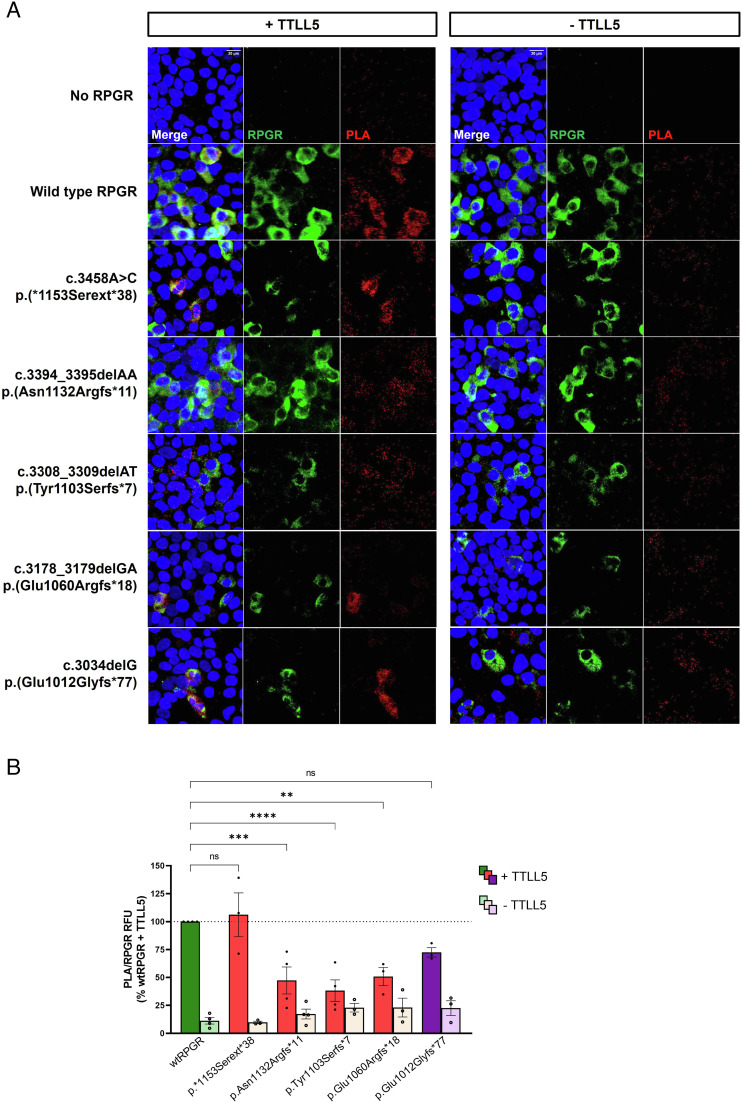
The frameshift and truncation of the RPGR^ORF15^ C-terminal basic domain disrupt the interaction with TTLL5. (*A*) Fluorescence micrographs of HEK293 cells transfected with human RPGR constructs in combination with TTLL5 expression plasmid (columns 1–3) and with only RPGR constructs (columns 4–6). The interaction between TTLL5 and RPGR was detected by in situ PLA (red, columns 3 and 6). The cells were counterstained with labeled FITC anti-RPGR antibody (green, columns 2 and 5) and DAPI (blue) to visualize the level of expression of RPGR and the nuclei, respectively. As negative controls, non-transfected HEK293 cells and HEK293 cells transfected only with TTLL5 (*Top* row) were included in the analysis. Scale bar: 20 µm. (*B*) Quantification of the PLA mean gray value normalized to the RPGR mean gray value in 6–10 single cells per sample, from at least, three independent experiments. The relative fluorescence units (RFU) is expressed as the percentage of the control (wtRPGR + TTLL5) (mean ± SEM). Significance was tested in samples co-transfected with RPGR and TTLL5 constructs by One-way ANOVA test followed by Dunnett’s multiple comparison test (**P < 0.01, ***P < 0.001, ****P < 0.0001, ns: nosignificant).

Wild-type RPGR and frameshift/truncated RPGR constructs were expressed alone or in combination with TTLL5. As shown in Fig. 6*A*, RPGR was expressed in the cytoplasm of cells co-transfected with RPGR WT and mutated constructs. Wild-type RPGR and TTLL5 showed clear co-localization in those cells expressing wtRPGR, represented by a high density of PLA puncta (red) per positive cell. RPGR construct p.*1153Serext*38, which is predicted to lead to a 38-amino acid extension of the wild-type RPGR protein, did not impair the binding to TTLL5 generating a high density of PLA puncta in RPGR-expressing cells. The RPGR constructs p.Asn1132Argfs*11 and p.Tyr1103Serfs*7 that led to abnormal C-terminal basic domain (basic domain sequence: 71.6% wild type, 14.9% altered, and 13.5% truncated; and 32.4% wild type, 9.5% altered, and 58.1% truncated, respectively), displayed markedly fewer interactions with TTLL5 compared with the wild type RPGR protein. The scattered PLA puncta observed in the fluorescence micrographs indicated that there was some level of interaction between these RPGR variants and TTLL5, although this was reduced compared to the wild-type RPGR protein. When the whole basic domain was affected in constructs p.Glu1060Argfs*18 (entire basic domain missing) and p.Glu1012Glyfs*77 (13.5% of the basic domain is altered and 86.5% truncated), although the interaction was also reduced, a considerable level was still detected in the presence of TTLL5. In these cases, not all cells expressing the RPGR constructs appeared to show interaction between the proteins. However, those cells that did express the RPGR and generated the PLA signal, presented a high density of puncta similar to that observed with wild-type RPGR. In the absence of TTLL5, low detectable levels of PLA signal were generated in cells expressing RPGR WT and mutants. The Human Protein Atlas, which contains information about expression profiles in 69 human cell lines, determines a normalized TTLL5 transcript expression value of 7.3 out of 40 in HEK293 cells. This basal TTLL5 gene expression in HEK293 cells could explain the PLA signal observed when cells are single transfected with RPGR. Indeed, one of the advantages of this assay is its high sensitivity that allows the detection of proteins expressed at very low levels. Non-transfected HEK293 cells were used as negative controls.

To prevent a biased analysis raised from differences in RPGR expression and transfection efficiencies, the PLA and RPGR fluorescence was quantified as the mean gray value in up to 10 single cells per sample from, at least, three independent experiments. The ratio PLA/RPGR (RFU) showed that RPGR constructs p.Asn1132Argfs*11, p.Tyr1103Serfs*7 and p.Glu1060Argfs*18 led to a significant disruption of its interaction with TTLL5 (*P* < 0.001, *P* < 0.0001 and *P* < 0.01 respectively) (Fig. 6*B*). These constructs affected the basic domain (28.4% affected: 14.9% altered and 13.5% truncated, 67.6% affected: 9.5% altered and 58.1% truncated, and 100% BD truncated respectively) and the altered protein structure generated by these three mutants was associated with cone or mixed cone–cone–rod dystrophy in our patient cohort. Mutation p.Glu1012Glyfs*77, predicted to affect the structure of the basic domain (100% affected: 13.5% altered and 86.5% truncated) demonstrated a strong tendency to decrease their binding to TTLL5, albeit the differences were not significant. The change in RPGR structure in this case was associated with a cone–rod phenotype.

### Truncating Distal RPGR^ORF15^ Variants Associated with Cone Dystrophies Lead to Impaired RPGR^ORF15^ Glutamylation.

To evaluate whether variants associated with cone dystrophies in the C-terminal domain of RPGR disrupt the glutamylase activity of TTLL5, Western blot analyses were carried out in HEK293 cells co-transfected with RPGR WT/mutant constructs and TTLL5, using the GT335 antibody to detect glutamylated proteins. The proximal missense variant c.350G>A p.Gly117Glu, predicted to lead to full length RPGR protein and associated with a rod–cone phenotype, was investigated as a control variant in this analysis.

**Fig. 7.**
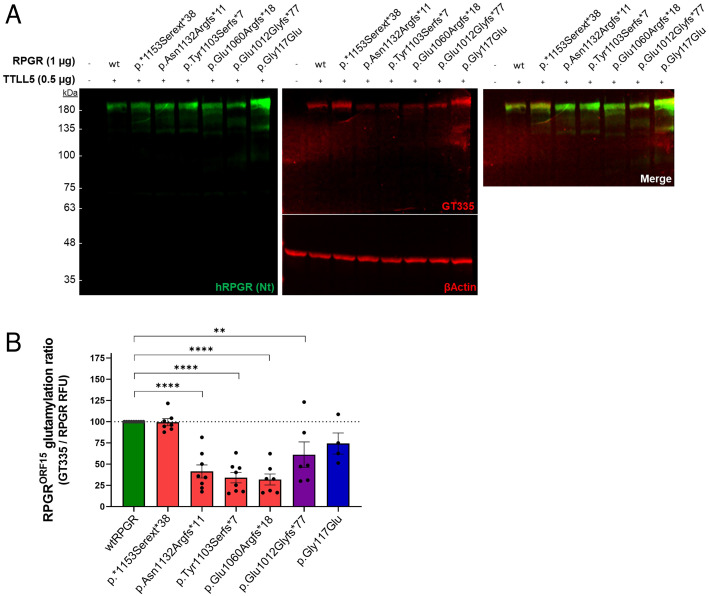
The level of RPGR^ORF15^ glutamylation is reduced when its C-terminal domain is truncated. (*A*) Whole protein lysates from transfected HEK293 cells were analyzed by SDS-PAGE and immunoblotting using an antibody against the N terminus (Nt) of the human RPGR (green) and the GT335 antibody (red). Anti-βActin (red, ca. 42 kDa) was used as an internal control. Non-transfected cells were used as negative control—only the βActin band is detected in this sample. Cells transfected with wild type and mutant forms of human RPGR^ORF15^ present a band at ca. 200 kDa, indicating RPGR protein (green). This band colocalizes with a GT335-reactive band (red) indicating that RPGR^ORF15^ is being glutamylated in all the transfected samples. (*B*) Quantification of the glutamylation ratio after normalizing to the RPGR protein levels. After confirming the normal distribution of the dataset (n = 4–9, Shapiro–Wilk and Kolmogorov–Smirnov tests), significance was tested by one-way ANOVA followed by Dunnett’s multiple comparison test (**P < 0.005, ****P < 0.0001).

First, we determined the optimal ratio RPGR/TTLL5 by transfecting HEK293 cells with increasing amounts of TTLL5-encoding plasmid. The expression of TTLL5, regardless of its levels, led to the glutamylation of wtRPGR (*SI Appendix*, Fig. S8). From this result, we decided to use the ratio 1:0.5 for our experimental analysis. Western blot analysis in whole protein lysates revealed a predominant band ca. 200 kDa, corresponding to the full length RPGR^ORF15^ protein, in all the cells transfected with RPGR construct, regardless of the variant (Fig. 7*A*). Glutamylation analysis in the same lysates revealed a GT335-immunoreactive band colocalizing with RPGR^ORF15^ protein. This result indicates that RPGR^ORF15^ is being glutamylated by TTLL5 in all the cells expressing RPGR. However, the intensity of the GT335-reactive bands was visibly lower in samples transfected with RPGR constructs p.Asn1132Argfs*11, p.Tyr1103Serfs*7, p.Glu1060Argfs*18, and p.Glu1012Glyfs*77, indicating that these mutants cause a reduction in the level of glutamylation of RPGR^ORF15^ protein in vitro. Fig. 7*B* shows the quantification of the glutamylation ratio, expressed as the GT335-band densitometry normalized to RPGR levels. This analysis indicates that glutamylation of RPGR^ORF15^ is significantly reduced (between 60 and 70%) in the frameshifted/truncated proteins associated with a cone-related phenotype, i.e., p.Asn1132Argfs*11 (total RPGR:0.9% altered/0.9% truncated; basic domain: 14.9% altered/13.5% truncated) (cone dystrophy), p.Tyr1103Serfs*7 (total RPGR:0.6% altered/3.7% truncated; basic domain: 9.5% altered/58.1% truncated) (cone dystrophy) and p.Glu1060Argfs*18 (total RPGR: 1.5% altered/6.5% truncated; basic domain: 100% truncated) (cone–cone–rod dystrophy) compared to wtRPGR (*P* < 0.0001). The difference in the level of glutamylation between wild-type RPGR^ORF15^ and RPGR p.Glu1012Glyfs*77 (total RPGR: 6.7% altered/5.5% truncated; basic domain: 13.5% altered/86.5% truncated) was also statistically significant (*P* < 0.005), although the reduction compared to wtRPGR was more moderate with this variant associated with the cone–rod phenotype without the central atrophy (39%). Glutamylation remained significantly unaffected when the length of the RPGR or the integrity of the basic domain were maintained as demonstrated with the p.*1153Serext*38 and p.Gly117Glu variants respectively.

## Discussion

Here we show that distal *RPGR^ORF15^* variants lead to cone-dominated RPGR phenotypes. In particular, the variants in the C-terminal basic domain are associated with a cone dystrophy phenotype with no apparent rod involvement. The truncating distal variants, associated with cone phenotypes, disrupt RPGR^ORF15^ interaction with TTLL5. Moreover, these variants significantly impair glutamylation of RPGR^ORF15^ by TTLL5. At the molecular level, this uncovers for the first time one of the pathogenic mechanisms of the cone dystrophy phenotype associated with distal RPGR variants which disrupt the integrity of the basic domain.

The *RPGR* gene is not unique in the heterogeneity of its phenotype and variants of other single genes have been associated with different retinal phenotypes. For example, biallelic loss-of-function variants in *PROM1* can lead to a pan-retinal disease, whereas missense variants can cause a cone dystrophy (41, 42). In this study, our genotype–phenotype correlations confirm the phenotypic spectrum of X-linked RGPR-related retinal disease. We find strong dependence of the RPGR phenotype on the variant location along the *RPGR* gene. The progressive shift from rod to cone-dominating phenotype as the *RPGR^ORF15^* variant location approaches the distal part of the ORF15 region is evident. The phenotype shift nonetheless is not a perfect continuum—we find a marked heterogeneity in the relative involvement of rod and cone photoreceptors in the rod–cone and cone–rod phenotype groups, in addition to the cone-only phenotype group. In our cohort, we did not, however, find a rod-only phenotype related to the RPGR disease, although we have previously reported a hypomorphic RPGR phenotype associated with an intronic variant which primarily affected the rods and where central cones and visual acuity remain relatively unaffected into the patient’s ninth decade (43). We also find that the rod photoreceptor contribution to the disease phenotype diminishes toward the C terminus, but that the rod–cone phenotype is found throughout the length of the RPGR, except in the basic domain. This substantially extends the previously reported “watershed region” of approximately 100 amino acids (949–1,047aa) (44) to the almost entire ORF15 region (676–1,047aa) where variants give rise to either rod-dominated or cone-dominated dystrophies. In addition, some pathogenic ORF15 variants (e.g., c.2405_2406del) lead to both rod-dominated and cone-dominated phenotypes, and we found that both phenotypes can exist within the same family, depicting the imperfect genotype–phenotype correlation. The reason for this variation remains unclear but could be due to the nature and extent of RPGR modifications and interactions with other cone or rod specific proteins, or indeed other genetic or environmental modifiers.

Cone involvement progresses from a relatively later onset, milder degeneration in rod–cone phenotypes at the proximal part of the gene, toward an earlier onset, more severe involvement in cone–rod dystrophies as the variants enter the ORF15 region and move toward the distal part of the gene. Indeed, the cone only dystrophy phenotype is mostly associated with variants which affect the very distal part of the ORF15 and the basic domain. In our study, we observed only one exception to this association, where a case of cone-dominated disease was associated with a more proximal truncating ORF15 variant p.Lys676Thrfs*17 (albeit with a milder phenotype compared with the distal cone variants), pointing to a more selective and mildly deleterious effect on foveal cones of this variant compared with other variants in the nearby location. The cone phenotype is unique in that it predominantly affects the foveal and parafoveal cones and generally has a more delayed onset with slow progression to central macular atrophy. There is no apparent rod photoreceptor involvement with this phenotype, even at the late-stage disease, as confirmed by electrophysiology recordings in our and other studies (38). However, our microperimetry studies show reduced retinal sensitivity in the area immediately surrounding the foveal atrophy, suggesting a more generalized macular cone dysfunction. The mean central retinal sensitivity is reduced in the early disease process across the RPGR phenotypes, including the rod-dominated dystrophies where the pattern is reversed (peripheral microperimetry loss with relative central foveal preservation), suggesting a primary involvement of cones in the RPGR disease, rather than a secondary phenomenon following rod degeneration. The central foveal atrophy of the cone dystrophy phenotype, was generally not a manifest feature in progressive rod–cone or cone–rod dystrophies until the end-stage pan-retinal degeneration or in the five cases of mixed cone–cone–rod phenotype where it developed in parallel to a more generalized cone and rod degeneration, suggesting a unique or additional molecular mechanism underlying the pathogenesis of cone dystrophies.

The mechanism of NMD is known to be a modulator of the phenotype of several genetic diseases (45, 46). In RPGR, the cutoff point for NMD is predicted to be between exons 14 and 15 – RPGR transcripts containing a mutation in exons 1–14 that create a premature translation termination codon are degraded by this cellular quality control system. Hence, the expression of RPGR protein in this case is reduced entirely and all the activities associated to this region (RCC1-like, protein–protein interactions, etc.) would be significantly reduced. This would have a predominant impact in rods, although cones would be ultimately affected (rod-dominated phenotype). If, however, frameshift mutations are located in the ORF15 region (escape NMD), a truncated RPGR protein that retains residual or partial activity is still synthetized, although the C-terminal sequence and structure will be compromised. In this case, the biological activity of the N-terminal domain is maintained, and this would suffice to ensure the survival of rods (at least for longer). The production of RPGR protein with truncated C-terminal domain would be more detrimental for cones, supporting our observations—truncating distal ORF15 variants impair glutamylation and this seems to affect cones more than rods (cone-dominated phenotypes). The genotype–phenotype correlations in this study indicate, indeed, a link between the observed RPGR phenotypes and the RPGR protein domains and their function in photoreceptors. Since the rod-dominated phenotypes affect the N terminus and involve the RCC1-like domain, the domain is likely to have a more important role in rods, whereas the ORF15 region predominantly associated with cone phenotypes is likely to serve a more dominant function in cones. We find the RRC1-like domain variants to be exclusively associated with the rod-dominated phenotypes and are thus likely to reduce the activity of the RCC1-like domain in the activation of small GTPases such as RAB8A (7) and/or the interaction with other specific proteins that play an essential role in rod photoreceptors such as PDE6δ (47, 48) or RPGRIP1 (49, 50). On the other hand, variants affecting the proximal part of the ORF15 which is rich in repetitive glycine and glutamic acid residues predominantly leads to a cone–rod phenotype. This repetitive sequence is normally likely to exist as a linear sequence bridging the globular N- and C-terminal domains and the variants which disrupt this region could affect the protein folding, including its stability leading to smaller amounts of protein, with a more detrimental effect on cone function (51). The high glutamic acid content of the ORF15 region is responsible for the negative charge associated to this domain, which is likely to mediate in the interaction between RPGR^ORF15^ and positively charged substrates, for example, scaffolding proteins that bind the negatively charged phospholipids of membranes. Mutations that change this negatively charged feature of RPGR^ORF15^ could affect its binding to protein complexes that are required for the homeostasis of rods and cones. The very distal variants which are not part of this repetitive and highly charged region of the ORF15 result in the cone dystrophy phenotype. Specifically in this study, we show that the highly conserved C-terminal part of the ORF15, the basic domain, has an essential function in the optimal functioning of cone photoreceptors. This is linked to the key protein interaction between RPGR^ORF15^ and TTLL5 which modify RPGR with post-translational glutamylation, possibly involved in the trafficking of cone opsins and other cargo via the connecting cilium to the photoreceptor outer segments (52). Thus, we find that the distal RPGR^ORF15^ variants which disrupt the integrity of the basic domain, through frameshifts and truncations, interfere with TTLL5 enzyme interaction. In turn, this results in reduced levels of glutamylated RPGR protein. Our results are in support of previous findings which demonstrate that RPGR^ORF15^ constructs which delete sections of the repetitive purine rich ORF15 region, or the entire length of the basic domain, significantly reduce glutamylation and lead to early mislocalization of cone opsins (17). Here we also show that truncating RPGR^ORF15^ variants, which affect only parts of the basic domain, also significantly impair the RPGR glutamylation and lead to the cone dystrophy phenotype. The significance of this finding reflects the heightened importance of RGPR^ORF15^/TTLL5 interaction and glutamylation to normal functioning of cones compared to rod photoreceptors. This could be related to the open lamellar membrane structure of cone outer segments sensitive to even minor mislocalizations of cone opsins or the differential gene expression profiles between rods and cones. In addition, higher metabolic demands associated with densely packed foveal cones could explain the preferential involvement of central foveal cones. It is therefore not surprising that in human retina, TTLL5 expression is more abundant in cones compared to rods (29). Moreover, we show that two constructs which do not affect the integrity of the basic domain, also do not impair RPGR glutamylation. Thus, c.350G>A variant located at the proximal part of the *RPGR* near the N terminus, which leads to a missense change in the RCC1-like domain but otherwise full length glutamylated RPGR results in a predominant rod phenotype, with minimal effect on cones. The second variant c.3458A>C, located at the very tip of the *RPGR* C terminus leaves the basic domain unaltered, but rather extends the normal length RPGR by 38 residues. Despite otherwise apparently unaffected RPGR structure, intact basic domain, and normal level of glutamylation which unsurprisingly preserve rod function, our study cannot exclude that the extended tail itself (via a dominant negative effect) negatively affects the folding and structure of RPGR protein preventing the binding to other proteins and eventually, interfering with the normal trafficking of cone opsins leading to the cone dystrophy, also observed with a similar RPGR extension (44). In addition, despite the overall normal levels of glutamylation found with this variant, the abnormal protein extension could lead to defects or aberrations in the glutamylated chains which could affect the folding and stability of the 3-D RPGR protein structure, its interactions with other key trafficking partners, the interaction of TTLL5 with its key regulators such as CSAP (53), or the balanced ratio between the glutamylation and other supportive post-translational modifications that could change the properties of RPGR protein such as glycylation (54). Furthermore, the additional effect of pathogenic variants on the balance between *RPGR^ORF15^*, constitutive *RPGR^1–19^* and other possible *RPGR* isoforms and their relative contribution to the disease phenotypes cannot be ruled out (55).

The molecular mechanisms behind the RPGR disease are complex. Our study furthers the insight into the pathogenesis of cone-dominated RPGR dystrophy associated with truncating distal ORF15 variants and links the phenotype to that caused by the loss of TTLL5 enzyme necessary for glutamylation and normal function of RPGR in retinal photoreceptors.

## Materials and Methods

The study design of this retrospective case series adhered to the tenets of the Declaration of Helsinki (56). Patients were identified from genetic databases at two clinical centers, Oxford, United Kingdom, and Bonn, Germany. Data on clinical assessments, retinal imaging studies (color photography, fundus autofluorescence, optical coherence tomography, wide-field fundus imaging), microperimetry and full-field electroretinography were collected. Sequence variations in *RPGR* were identified by targeted next-generation sequencing techniques and putative pathogenic variants were confirmed by Sanger sequencing. The ORF15 region of the RPGR gene was analyzed in all cases. In vitro molecular assays were performed to assess the impact of mutations in the C-terminal domain of RPGR protein on its interaction with TTLL5, and its level of glutamylation. Methodological details are provided in **SI Appendix*, SI Materials and Methods*.

## Supplementary Material

Appendix 01 (PDF)

## Data Availability

All study data are included in the article and/or *SI Appendix*.
