# Supplementary material for: Impaired glutamylation of RPGRORF15 underlies the cone-dominated phenotype associated with truncating distal ORF15 variants
Source: Proc Natl Acad Sci U S A. 2022 Nov 29;119(49):e2208707119. doi: 10.1073/pnas.2208707119 (PMC9897430; doi:10.1073/pnas.2208707119)
Supplement: Supplementary file 1 — Appendix 01 (PDF) [file pnas.2208707119.sapp.pdf]

PNAS
www.pnas.org

Supplementary Information for

Impaired glutamylation of RPGR^{ORF15} underlies the cone dominated phenotype associated with truncating distal ORF15 variants

*Jasmina Cehajic-Kapetanovic[1,2, †], *Cristina Martinez-Fernandez de la Camara[1,2, †], Johannes Birtel[3], Salwah Rehman[1,2], Michelle E McClements[1,2], Peter Charbel Issa[2,3], Andrew J Lotery[4,5], Robert E MacLaren[1,2, †].

[1]Nuffield Laboratory of Ophthalmology, Department of Clinical Neurosciences, John Radcliffe Hospital, Level 5 & 6, West Wing, Headley Way, OX3 9DU, UK

[2]Oxford Eye Hospital, Oxford University Hospitals NHS Trust, John Radcliffe Hospital, West Wing, Headley Way, OX3 9DU, UK

[3]Department of Ophthalmology, University of Bonn, Bonn, Germany

[4]Clinical Neurosciences Research Group, Clinical and Experimental Sciences, Faculty of Medicine, University of Southampton, Southampton, UK.

[5]University Hospital Southampton NHS Foundation Trust, Southampton, UK

*Contributed equally to this work

† Corresponding authors: Jasmina Cehajic-Kapetanovic, Cristina Martinez-Fernandez de la Camara, Robert E MacLaren (enquiries@ndcn.ox.ac.uk)

This PDF file includes:

SI Materials and Methods
Figures S1 to S8
Table S1
References

SI Materials and Methods

Clinical Assessments

The study design of this retrospective case series adhered to the tenets of the Declaration of Helsinki (1). Patients were identified from genetic databases between May 1, 2018, and February 1, 2022, at 2 clinical genetic centres in Oxford, United Kingdom, and Bonn, Germany. Institutional review boards at the study centres approved the studies, and patients provided written informed consent. Patients received no compensation or incentive to participate. The data analysis was conducted from May 1, 2019, to February 1, 2022. Medical records of patients with RPGR variants were reviewed for information on family, general history, and ophthalmic history. Details of clinical assessments, including visual acuity and dilated fundal examinations, were collected. Retinal imaging studies, including colour photography, fundus autofluorescence (55 and 30), and optical coherence tomography (OCT), were taken with the Heidelberg Spectralis system (Heidelberg Engineering, Heidelberg, Germany). In addition, widefield fundus imaging was performed with the Optos 200Tx confocal scanning laser ophthalmoscopy camera (Optos, Dunfermline, UK) and central retinal sensitivity was measured using MAIA microperimetry (MAIA, CenterVue, Padova, Italy). In cases where microperimetry was not possible/recordable due to advanced disease (16 patients), a zero score was assigned. These patients are marked NP in Fig S2. Full field and pattern electroretinography (ERG) were performed according to the International Society for Clinical Electrophysiology of Vision standard.

Sequence variations in RPGR were identified by targeted next-generation sequencing techniques as previously described, involving analysis of 115 genes associated with RP and RP-like phenotypes. Putative pathogenic variants were confirmed by Sanger sequencing. The ORF15 region of the RPGR gene was analysed in all cases. The pathogenicity of identified variants was assessed in a population-based genome data set (the Single Nucleotide Polymorphism Database). The allelic frequency of the variants was further evaluated in gnomAD, which includes the Exome Aggregation Consortium data set.

Generation of human RPGR mutants

$RPGR^{ORF15}$ mutant constructs were generated by site-directed mutagenesis (QuickChange II XL kit, Agilent) using as a template a construct that expresses the wild type (WT) $RPGR^{ORF15}$ protein,

under the control of the chicken β-actin (CAG) promoter. To express TTLL5, a clone expressing the complete TTLL5 ORF was acquired from Origene (#RC211990).

Cell culture and transfection

HEK293 cells were maintained in DMEM (Gibco) with 10% heat inactivated foetal bovine serum (FBS) and 1% penicillin/streptomycin at 37°C and 5% CO_2. Transient transfection was performed using the Mirus TransIT®-LT1 Transfection Reagent (Geneflow Ltd., Lichfield, UK) in serum/antibiotic free medium, following manufacturer's instructions. Cells were transfected with 0.2 μg/cm^2 RPGR constructs alone or in combination with 0.1 μg/cm^2 TTLL5. Transfected cells were incubated at 37°C for 48 hours.

Western Blot

Forty-eight hours after transfection, the cells were lysed in lysis buffer containing protease inhibitors and spun at 14,000 x g for 10 min at 4 °C. Sixty μg of total protein was denatured in 6 x Laemmli buffer (Sigma-Aldrich) for 5 minutes at 95°C and separated on a 7.5% sodium dodecyl sulfate polyacrylamide gels (CriterionTM TGXTM Precast Gels, Bio-Rad Laboratories Ltd., Hemel Hempstead, UK) for electrophoresis at 100 V for 2 hours. Protein samples separated in the SDS-PAGE were transferred onto polyvinylidene difluoride (PVDF) membranes (Trans-Blot Turbo™ Midi PVDF, Bio-Rad) using the Trans-Blot Turbo™ Transfer Starter System (Bio-Rad). The membranes were blocked with 5% non-fat milk in Tris-buffered saline containing 0.1% Tween-20 (TBS-T) for 60 min at room temperature, and incubated with primary antibodies overnight at 4 °C. To assess the level of glutamylation, the GT335 antibody (Cat no. AG-20B-0020-C100, AdipoGen, San Diego, CA) was used (1:300 dilution). To identify the comigration of GT335-reactive bands with human RPGR, a human RPGR antibody raised against the N-terminus (Cat no. HPA001593, Sigma-Aldrich) was used (1:300 dilution). Mouse monoclonal anti-βactin antibody obtained from Thermo Scientific (Cat no. AM4302) was used as loading control (1:1,000 dilution). Bands were detected with fluorescent IRDye secondary antibodies (LiCor). Image Studio Lite (v5.2, Li-Cor Biosciences) was used for quantitation of the abundance of proteins on western blot images.

Proximity Ligation Assay and immunofluorescence

To evaluate the interaction between the different RPGR constructs and TTLL5, a proximity ligation assay (PLA) was carried out with Duolink® In Situ Detection Reagents Red (Sigma Aldrich) following manufacturer's instructions. Briefly, cells were fixed in 4% paraformaldehyde in PBS for 10 min and permeabilized with 0.2% Triton X-100 in PBS at room temperature for 5 min. Cells were

washed three times with PBS and blocked with Blocking solution for 60 min at 37 °C. Since the two primary antibodies were raised in rabbit, the Duolink® In Situ Probemaker PLUS and MINUS (Sigma Aldrich) were used to conjugate the oligo arms directly to the primary antibodies. Cells were incubated with the primary antibodies conjugated to the PLA probes for 2h at room temperature. After washing with Duolink® In Situ Wash Buffers for fluorescence (Sigma Aldrich), the slides were incubated with the DNA ligase for 30 min at 37 °C. Slides were washed and incubated with a DNA polymerase for 100 min at 37 °C. A counterstaining step was performed by incubating the slides with labeled FITC anti-RPGR antibody for 1h at room temperature. Slides were washed and mounted with a coverslip using Duolink® In Situ Mounting medium with DAPI (Sigma Aldrich). Images were acquired using the Zeiss LSM-710 inverted confocal microscope system (Zeiss, Oberkochen, Germany) with a pinhole setting of 1 airy unit. Three different fields per sample were acquired with the 40x objective and at 2.2x zoom under standardized conditions of ambient lighting, gain and exposure. Fluorescence was quantified using ImageJ software. PLA and RPGR mean gray value of single cells expressing RPGR was quantified using the freehand selection and ROI manager tools.

Statistical analysis

GraphPad Prism (version 9.3.1, GraphPad Inc., USA) was used for statistical analysis. To prove for significance of observed differences, non-parametric data was analyzed using the Kruskal-Wallis test followed by Dunn's multiple comparison test. When normal distribution of the dataset was confirmed by Shapiro-Wilk and Kolmogorov-Smirnov tests, significance was tested by one-way ANOVA followed by Dunnett's multiple comparison test. P-value of 0.05 and below was considered to be significant.

Supplemental figures

FIG. S1: Progression of the different RPGR-associated phenotypes with age

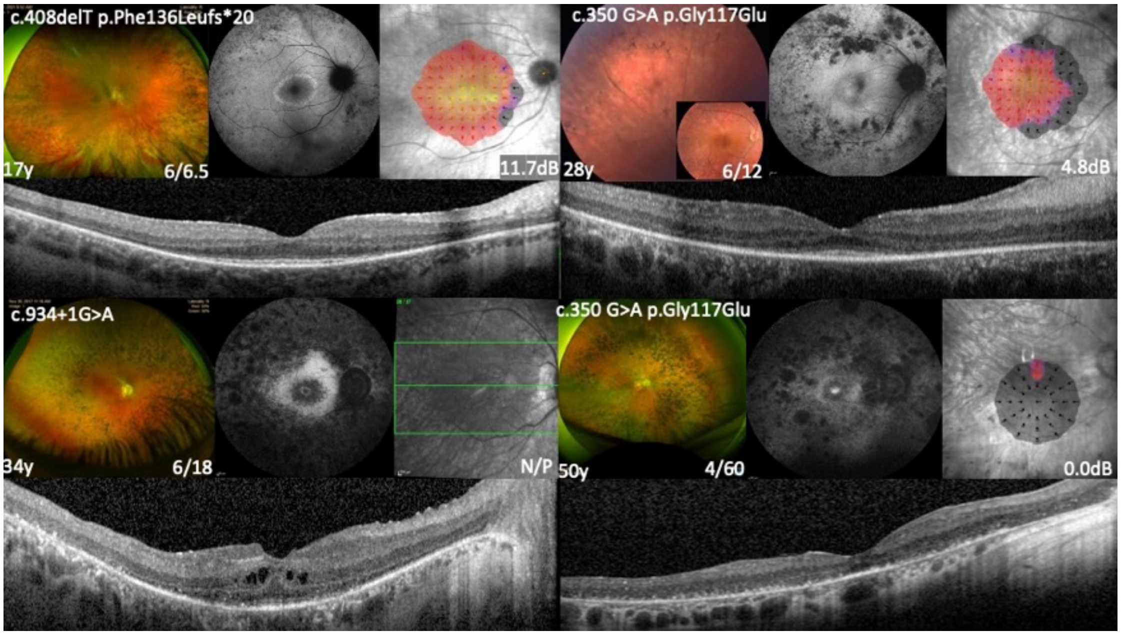

Fig S1A. Rod-cone phenotype progression with age. Early disease stage shows predominant rod degeneration in periphery with pigment migration, some reduction in central retinal sensitivity but preserved visual acuity. Later, there is increase in peripheral rod degeneration with more pigmentation and marked patchy peripheral hypo-autofluorescence. In addition, cone degeneration and dysfunction also occur with increasing loss of central retinal sensitivity and visual acuity.

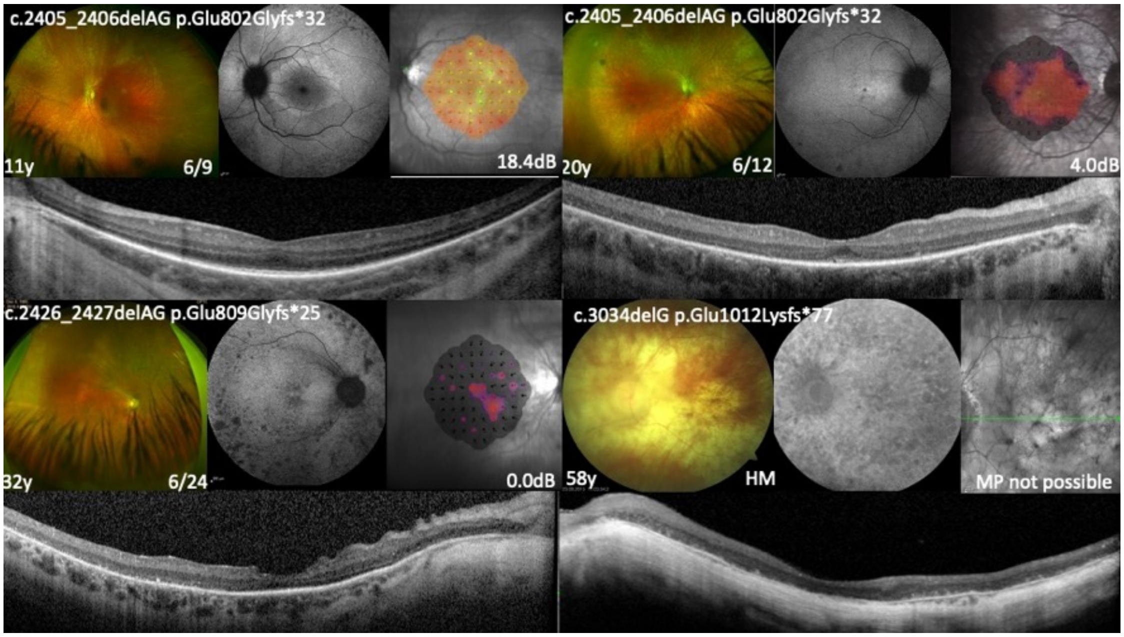

Fig S1B. Cone-rod phenotype progression with age. Early disease stage shows mild reduction of central cone function with already affected visual acuity. Later, progressive cone degeneration is evident with disruption of central ellipsoid zone, increased loss of retinal sensitivity and marked loss of visual acuity. There is however relatively mild to moderate rod degeneration with minimal pigmentary retinopathy in periphery even at very advanced degeneration.

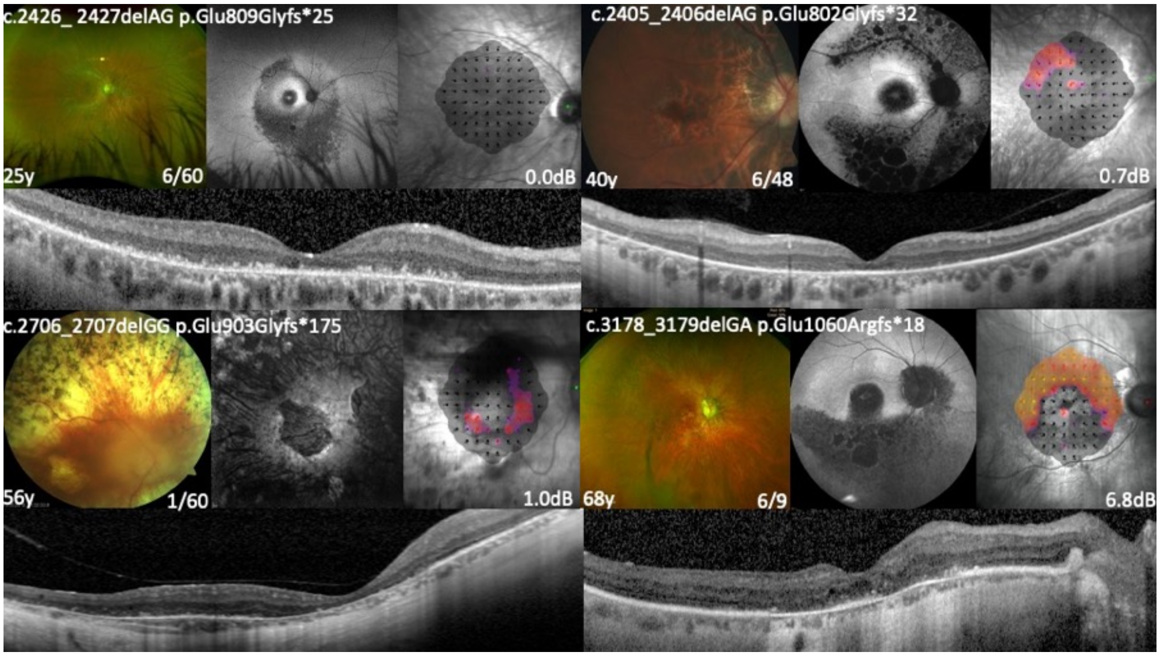

Fig S1C. Cone/Cone-Rod phenotype progression with age. Even at early stages, there is marked cone dystrophy and central cone dysfunction accompanied with mid-peripheral (or sectoral) rod degeneration marked by hypo-autofluorescent patches. Later, there is progressive macular atrophy (foveal sparing in some cases), central reduction in retinal sensitivity and in some late-stage cases pigmentary retinopathy.

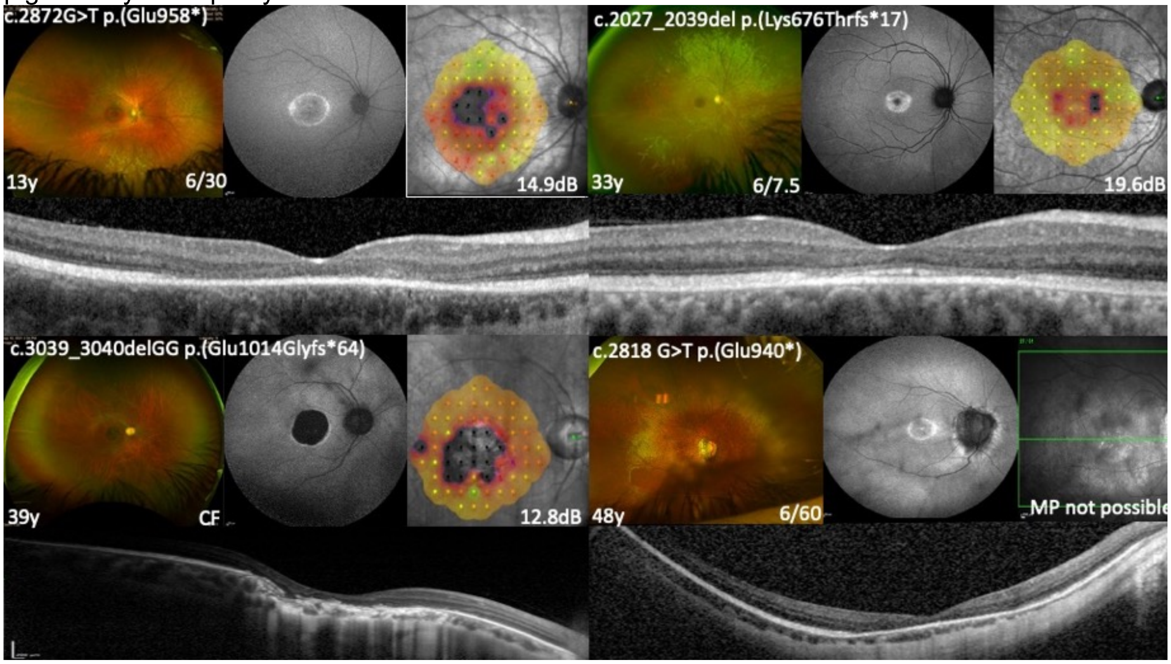

Fig S1D. Cone dystrophy phenotype progression with age. Early stages show central macular dystrophy with central scotoma and markedly reduced visual acuity in most cases. There is no apparent rod-related degeneration or peripheral pigmentary retinopathy even at late-stage degeneration.

FIG. S2: BCVA and microperimetry plots for our RPGR patient cohort related to the variant location along the RPGR

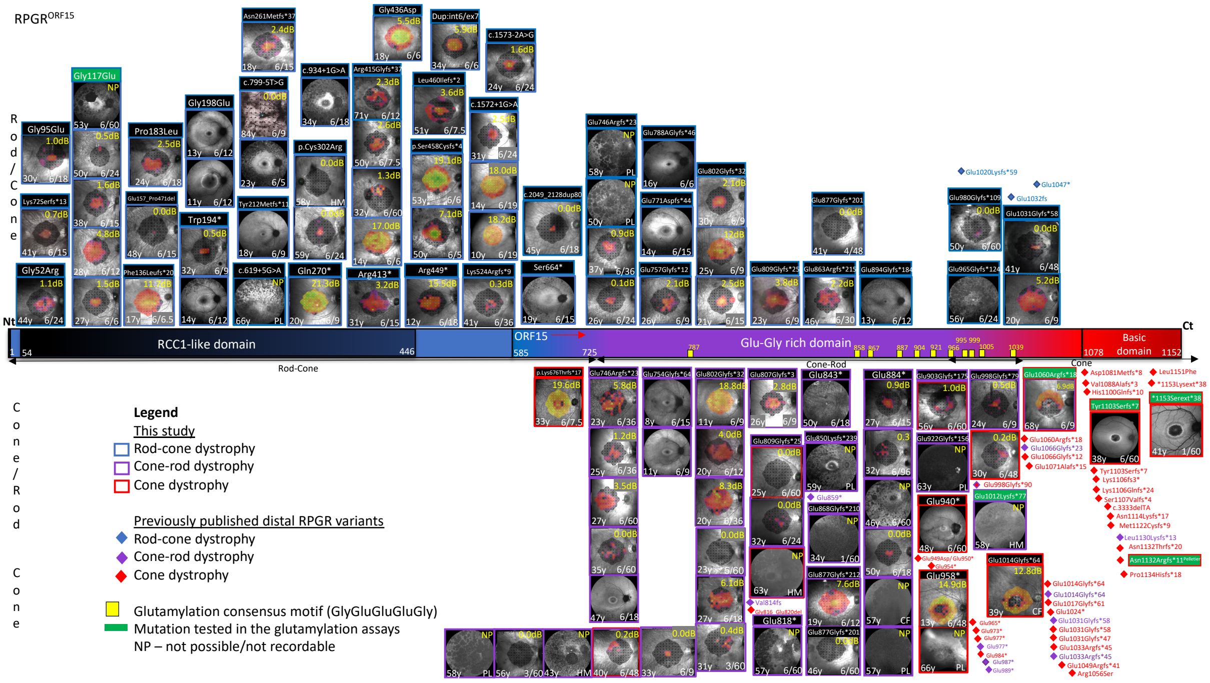

Fig S2. Microperimetry plots showing reduced central retinal sensitivity with all phenotypes even at early stage of disease when visual acuity can be normal or very mildly reduced.

FIG. S3: BCVA and microperimetry data for our RPGR patient cohort related to the clinical phenotype, the variant location and patients' age.

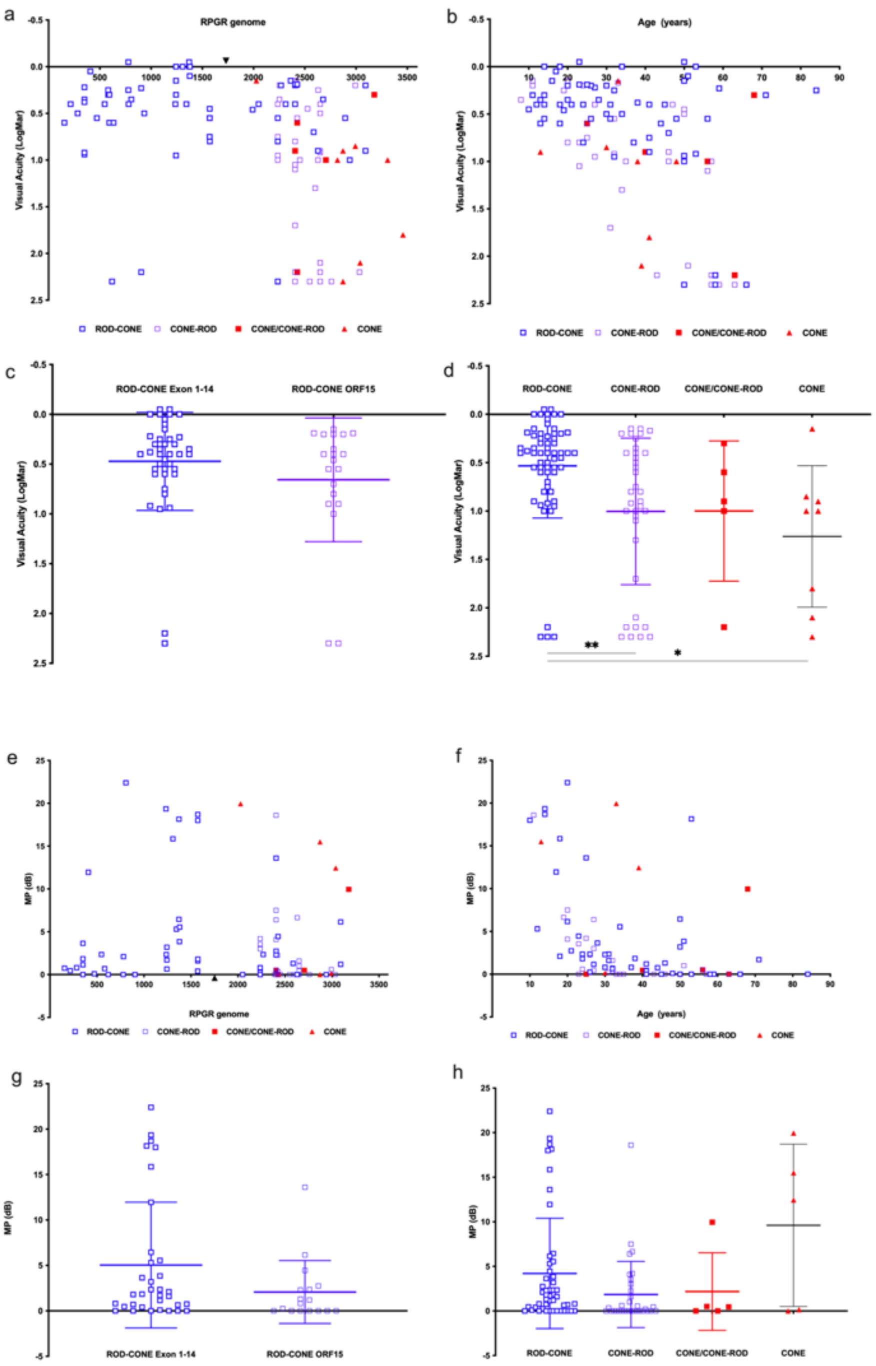

Fig S3. Variant location along the RPGR genome leads to progressive shift from rod to cone phenotype affecting visual acuity and microperimetry with advancing disease. Cohort distribution of visual acuity along the RPGR gene (a) or patient's age (b) according to the RPGR disease phenotype. Mean visual acuity for rod-cone variants in exons 1-14 versus ORF15 (c) and each RPGR disease phenotype (d). Cohort distribution of microperimetry along the RPGR gene (e) or patient's age (f) according to the RPGR disease phenotype. Mean microperimetry for rod-cone variants in exons 1-14 versus ORF15 (g) and each RPGR disease phenotype (h). (^) marks the ORF15 breakpoint. Following tests for normal distribution, significance was tested by one-way ANOVA and Kruskal-Wallis test followed by Dunn's multiple comparisons test or Mann-Whitney test (*p<0.05).

FIG. S4: BCVA data for our RPGR patient cohort related to the clinical phenotype and the age group.

a

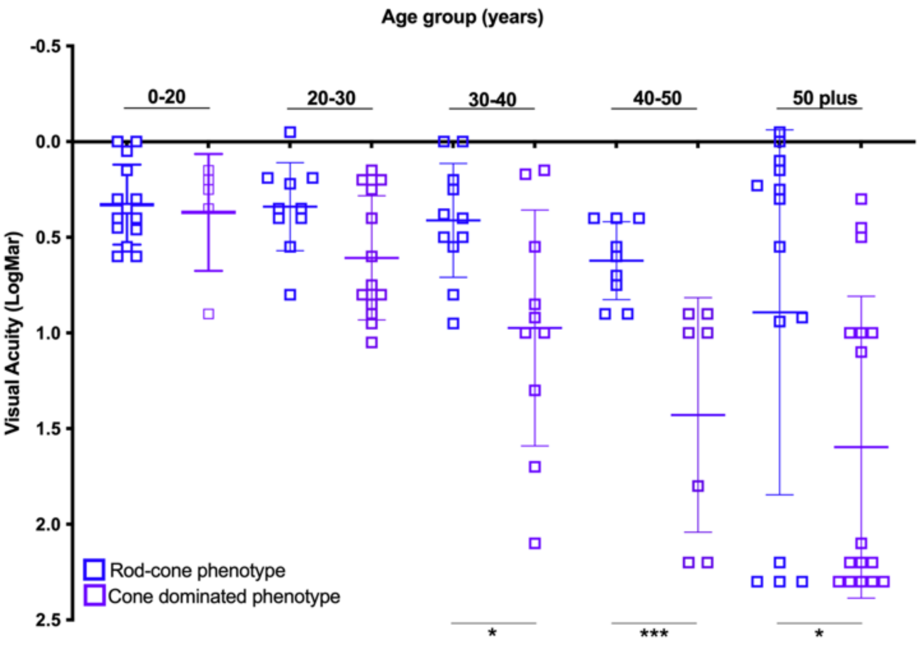

b

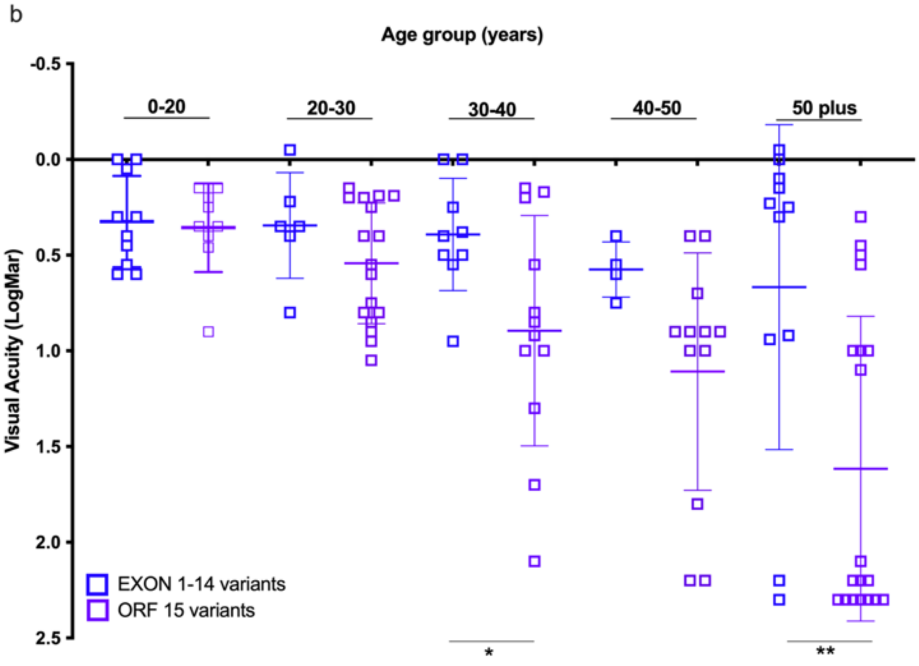

FIG S4. Mean visual acuity for rod-cone versus cone-dominated phenotypes at different age groups (a). Mean visual acuity for variants in exons 1-14 versus ORF15 at different age groups (b). Following tests for normal distribution, significance for each age group was tested by Mann-Whitney test (*p<0.05).

FIG. S5: Microperimetry data for our RPGR patient cohort related to the clinical phenotype and the age group.

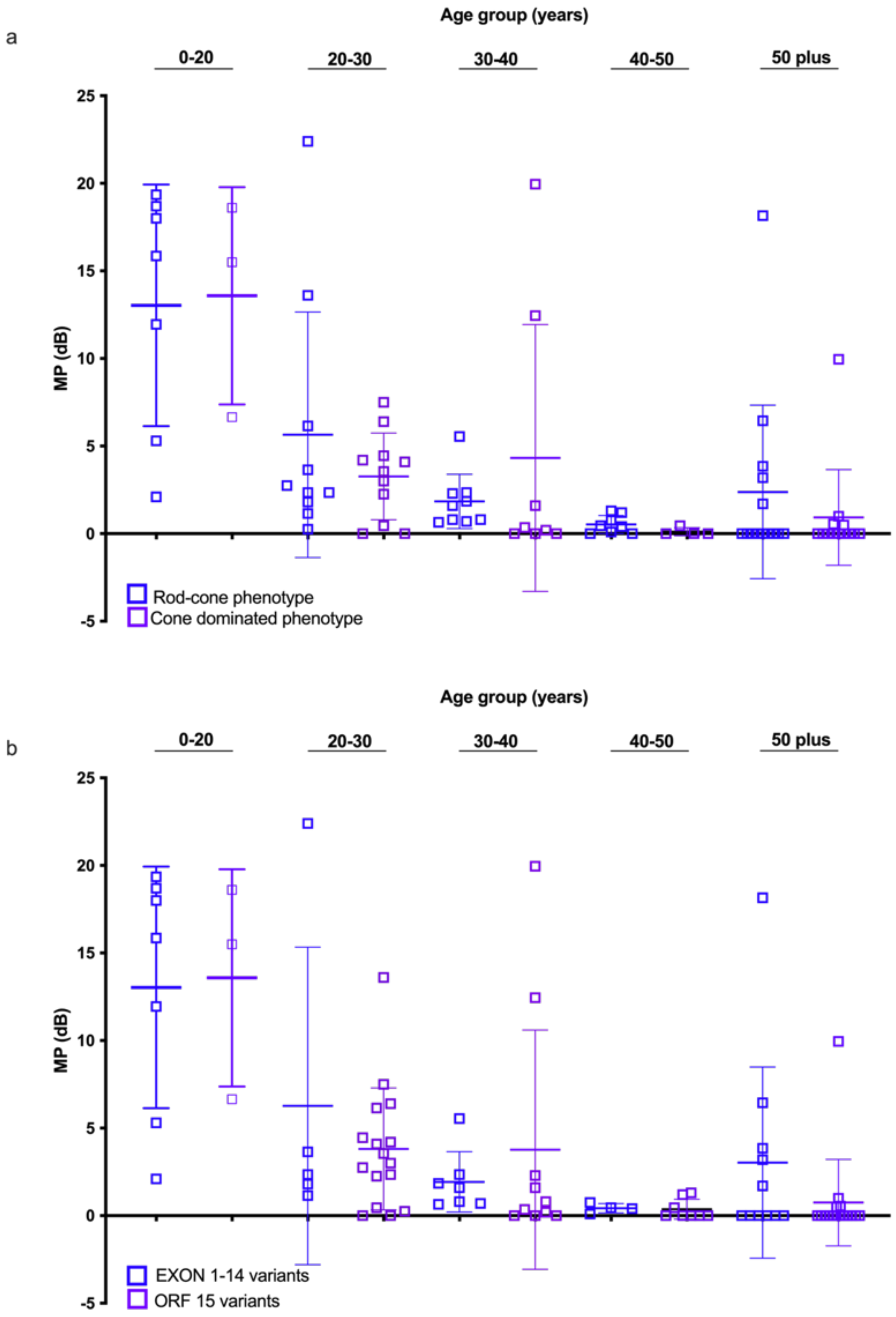

FIG S5. Mean microperimetry for rod-cone versus cone-dominated phenotypes at different age groups (a). Mean microperimetry for variants in exons 1-14 versus ORF15 at different age groups (b). Following tests for normal distribution, significance for each age group was tested by Mann-Whitney test (*p<0.05).

FIG. S6: TTLL5 associated retinal dystrophy phenotype

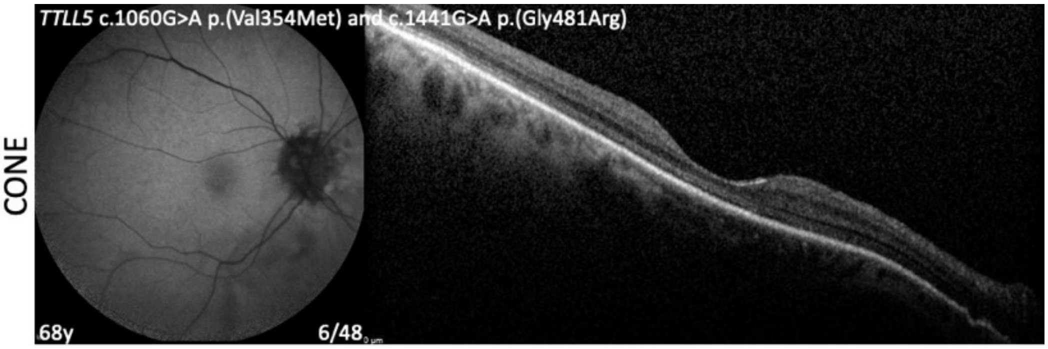

Fig S6. Cone dystrophy phenotype associated with heterozygous *TTLL5* variants (c.1060G>A and c.1441G>A). Fundus autofluorescence image (left) shows decreased perifoveal autofluorescence. SD-OCT (right) shows major thinning of the outer nuclear layer at the macula with marked disruption/loss of the ellipsoid zone.

FIG. S7: Nonsense-mediated decay prediction for RPGR truncated variants

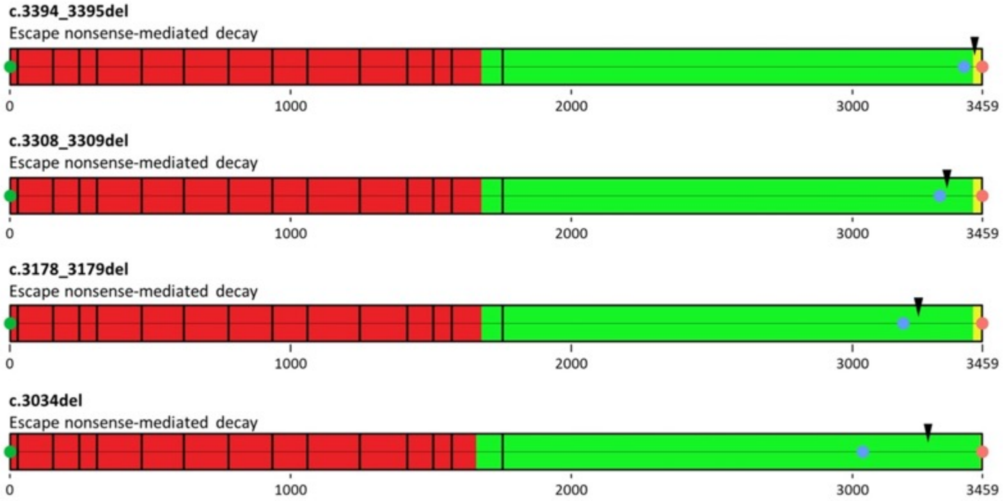

Fig S7. Non-sense-mediated decay (NMD) prediction of all the *RPGR*-truncating variants included in the glutamylation assays. Green circle indicates the CDS start site; red circle indicates the canonical stop site of RPGR protein; blue circle indicates the location of the truncating variant. Black arrow symbol indicates the predicted termination site of the mutated protein. The region in which truncating variants lead to NMD is indicated in red, whilst the alleles with truncating variants located in the green part of protein could escape from NMD. The variants located in the yellow region of the protein may lead to non-stop RNA decay. The four RPGR transcripts harboring frameshift mutations analyzed in the glutamylation assays are predicted to escape NMD.

FIG. S8: Western blot analysis showing RPGR WT glutamylation by TTLL5.

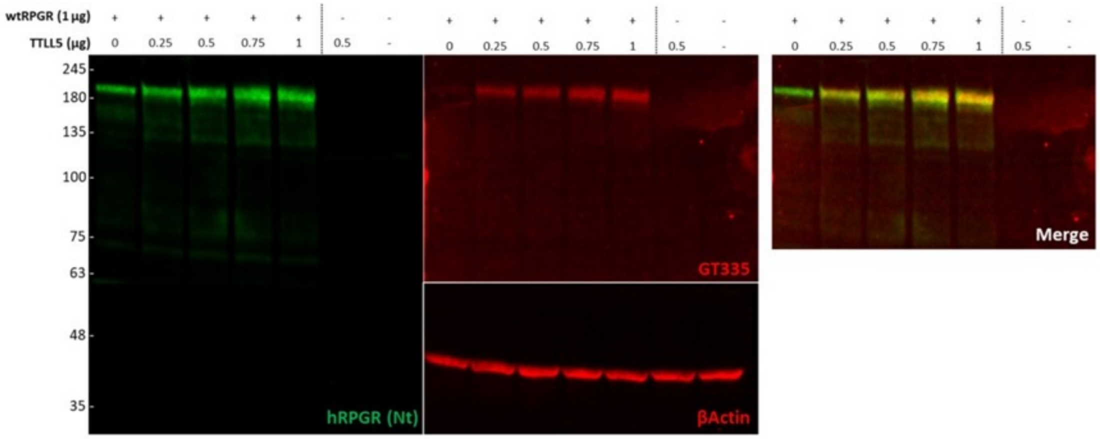

Fig S8. Wild type RPGR^{ORF15} is glutamylated by TTLL5. Whole protein lysates from HEK293 cells transfected with 1 µg wtRPGR plasmid and increasing amounts of TTLL5 plasmid (0.25, 0.5, 0.75 and 1 µg) were analyzed by SDS-PAGE and immunoblotting using anti-RPGR (green) and the GT335 antibody (red). Anti-βActin (red, ca. 47 kDa) was used as an internal control. Non-transfected cells, and cells single transfected with 0.5 µg of TTLL5 were used as negative control - only the βActin band is detected in these samples. Cells transfected with wild type human RPGR^{ORF15} present a band at ca. 200 kDa, indicating RPGR protein (green). In samples expressing TTLL5, RPGR band colocalizes with a GT335-reactive band (red), indicating that RPGR is being glutamylated by TTLL5.

Supplemental Table 1: Electrophysiology studies

Table S1. Results of electrophysiology (ERG) recordings from patients with *RPGR* phenotypes: rod-cone, cone-rod, cone/cone-rod and cone and a patient with a *TTLL5* cone phenotype.

ID	Age (y)	*RPGR* variant	Phenotype	Visual Acuity (logMar)	Scotopic ERG (% lower limit)	Photopic ERG (% lower limit)	Photopic 30Hz flicker	Pattern ERG
#1	14	c.154G>A p.Gly52Arg	rod-cone	0.7	35.4	50	Detected (21μV@44ms)	Not recorded
#2	30	c.284G>A p.Gly95Glu	rod-cone	0.5	0	0	Undetectable	Not recorded
#3	18	c.633delA p.Tyr212Metfs*11	rod-cone	0.6	0	10	Detected (5μV@41ms)	Not recorded
#4	12	c.1345C>T p.Arg449*	rod-cone	0.4	0	7	Detected (2μV@27ms)	Not recorded
#5	14	c.1572+1G>A	rod-cone	0.55	0	2	Detected (3μV@18ms)	Not recorded
#6	10	c.1572+1G>A	rod-cone	0.45	0	4	Detected (2μV@29ms)	Not recorded
#7	19	c.1991C>A p.Ser664*	rod-cone	0.45	0	0	Undetectable	Not recorded
#8	26	c.2236_2237delGA p.Glu746Argfs*23	rod-cone	0.55	0	0	Detected (2μV@20ms)	Not recorded
#9	16	c.2363_2364delAG p.Glu788Glyfs*46	rod-cone	0.15	0	0	Detected (7.5μV@37ms)	Not recorded
#10	21	c.2405_2406delAG p.Glu802Glyfs*32	rod-cone	0.4	0	0	Detected (2.5μV@26ms)	Not recorded
#11	30	c.2405_2406delAG p.Glu802Glyfs*32	rod-cone	0.2	0	14	Detected (5μV@38ms)	Undetectable
#12	24	c.1573-2A>G	rod-cone	0.8	37	54	Detected (24.8μV@34ms)	Not recorded
#13	8	c.2253_2260dup p.Glu754Glyfs*64	cone-rod	0.35	0	0	Undetectable	Not recorded
#14	11	c.2253_2260dup p.Glu754Glyfs*64	cone-rod	0.2	0	0	Undetectable	Not recorded
#15	20	c.2405_2406delAG p.Glu802Glyfs*32	cone-rod	0.15	5	0	Undetectable	Undetectable
#16	25	c.2405_2406delAG p.Glu802Glyfs*32	cone-rod	0.19	64	6	Detected (11μV@40ms)	Recorded (1.9 & 2μV @63 ms)
#17	57	c.2452G>T p.Glu818*	cone-rod	1	4	6	Undetectable	Undetectable
#18	46	c.2628_2629delGG p.Glu877Glyfs*201	cone-rod	1	0	0	Undetectable	Undetectable
#19	19	c.2630delA p.Glu877Glyfs*212	cone-rod	0.25	19	1.3	Undetectable	Not recorded
#20	27	c.2650G>T p.Glu884*	cone-rod	0.4	28	20	Detected (17μV@40ms)	Recorded (0.6 & 1.4μV @63ms)
#21	63	c.2426_2427delAG p.Glu809Glyfs*25	cone/cone-rod	2.2	0	0	Undetectable	Undetectable
#22	68	c.3178_3179delGA p.Glu1060Argfs*18	cone/cone-rod	0.3	89	20	Undetectable	Undetectable
#23	33	c.2027_2039del12 p.Lys676Thrfs*17	cone	0.15	100	4	Undetectable	Undetectable
#24	13	c.2872G>T p.Glu958*	cone	0.9	100	5	Undetectable	Undetectable
#25	39	c.3039_3040delGG p.Glu1014Glyfs*64	cone	2.1	96	14	Detected (5μV@41ms)	Undetectable
#26	68	c.1060G>A p.Val354Met c.1441G>A p.Gly481Arg	*TTLL5* cone	0.9	100	67	Detected (36μV@28ms)	Undetectable

References

1. General Assembly of the World Medical A (2014) World Medical Association Declaration of Helsinki: ethical principles for medical research involving human subjects. *J Am Coll Dent* 81(3):14-18.
